# Improved brain community structure detection by two-step weighted modularity maximization

**DOI:** 10.1371/journal.pone.0295428

**Published:** 2023-12-08

**Authors:** Zhitao Guo, Xiaojie Zhao, Li Yao, Zhiying Long

**Affiliations:** School of Artificial Intelligence, Beijing Normal University, Beijing, China; ’Enrico Fermi’ Research Center, ITALY

## Abstract

The human brain can be regarded as a complex network with interacting connections between brain regions. Complex brain network analyses have been widely applied to functional magnetic resonance imaging (fMRI) data and have revealed the existence of community structures in brain networks. The identification of communities may provide insight into understanding the topological functions of brain networks. Among various community detection methods, the modularity maximization (MM) method has the advantages of model conciseness, fast convergence and strong adaptability to large-scale networks and has been extended from single-layer networks to multilayer networks to investigate the community structure changes of brain networks. However, the problems of MM, suffering from instability and failing to detect hierarchical community structure in networks, largely limit the application of MM in the community detection of brain networks. In this study, we proposed the weighted modularity maximization (WMM) method by using the weight matrix to weight the adjacency matrix and improve the performance of MM. Moreover, we further proposed the two-step WMM method to detect the hierarchical community structures of networks by utilizing node attributes. The results of the synthetic networks without node attributes demonstrated that WMM showed better partition accuracy than both MM and robust MM and better stability than MM. The two-step WMM method showed better accuracy of community partitioning than WMM for synthetic networks with node attributes. Moreover, the results of resting state fMRI (rs-fMRI) data showed that two-step WMM had the advantage of detecting the hierarchical communities over WMM and was more insensitive to the density of the rs-fMRI networks than WMM.

## Introduction

The human brain can be regarded as a complex network with interacting connections between brain regions. Complex brain network analyses have been widely applied to functional magnetic resonance imaging (fMRI) data, especially resting state fMRI (rs-fMRI), to reveal the neural mechanisms of various cognitive processes and brain diseases. Numerous studies suggest the existence of community structures in brain networks [1–3]. Communities are groups of nodes that have dense within-group connections and sparse between-group connections. Human brain consists of several segregated subsystems (communities) with specialized cognitive functions. Moreover, multiple subsystems often integrated with each other to participate in high-level cognitive processing. A hierarchical-modular brain network organization [4] is particularly well suited to accommodate diverse levels of segregated/integrated activity across many scales. Therefore, the community structures of brain networks favor a balance between segregation and integration of brain function, which plays a vital role in enhancing the robustness against outside attacks and improving the efficiency of information segregation and integration [3, 5–7].

Various community detection methods, mainly including spectral clustering [8], Infomap [9], Bayesian Community Detection (BCD) [10], the weighted stochastic block model (WSBM) [11] and modularity maximization (MM) [12], have been widely applied to neuroimaging data to reveal the community structure of brain networks [13–17]. However, all community detection methods above suffer from instability to various degrees, especially when the network scale is very large [18, 19]. For spectral clustering, BCD and WSBM, prior knowledge is needed to set the number of communities. Moreover, BCD and WSBM require setting of the models’ hyperparameters and assume an exponential family distribution of network edges. Among the above community detection methods, the MM method has the advantages of model conciseness, fast convergence and strong adaptability to large-scale networks and has been extended from single-layer networks to multilayer networks to investigate the community structure changes of brain networks in development [20], aging [17], diseases [21] and cognitive processes [22].

The MM method partitions the network nodes into nonoverlapping communities to maximize an objective function known as the modularity proposed in [23]. Blondel et al. proposed a heuristic MM method called the “Louvain heuristic” to extract the community structure of large-scale networks [12] and substantially accelerate the solving process. Mucha et al. extended the MM method from a single-layer network to a multilayer network and proposed a multilayer MM framework [24]. Moreover, the MM method can be extended to node-attributed networks by using simultaneous fusion or late fusion [25]. Some studies adopted simultaneous fusion methods that modified the structure-aware objective function of Louvain in MM by including an attributes-aware objective function in the optimization function [26]. For the late fusion methods, community partitions for structure were first separately performed by MM and attributes. Then, the partitions were fused to obtain the resulting structure- and attributes-aware partition [27, 28].

The solutions of MM suffer from instability because the MM method is nondeterministic and has many local maxima [17, 29]. Bassett et al. developed a method for constructing a robust partition by performing MM on the thresholded association matrix whose elements pass the statistical testing in comparison to null models and demonstrated that the statistical corrected association matrix showed stable community partitions for multiple runs of MM [30]. However, different null models may affect the thresholded association matrix. We call Bassett’s method robust MM for convenience in this paper.

Moreover, the MM method fails to detect hierarchical community structures [31] in networks. Multiresolution modularity was proposed to uncover communities of different sizes by introducing a tunable resolution parameter *γ* [32]. However, multiresolution modularity cannot reveal hierarchical community structure. Ruan et al. (2008) performed MM twice to subdivide the communities obtained from the MM in the first time [33], which could reveal the two-level hierarchical community structure. It was assumed that all the communities detected by the MM method contained sub-communities in Ruan’s study. However, such assumption may not be correct for all networks.

The above two problems of MM largely limit the application of MM in the community detection of fMRI data. In this study, we proposed the weighted modularity maximization (WMM) method that weighted the adjacency matrix by using the association matrix to improve the stability of MM. Many studies have shown that brain networks have hierarchical community structures [15, 34]. Based on WMM, we further proposed the two-step weighted modularity maximization (two-step WMM) method that can detect the hierarchical communities by judging whether the communities detected by MM is hierarchical structure through the node attributes. The two-step WMM method further subdivided the communities obtained from the first WMM step if the attribute distance between nodes within the community was significantly reduced after subdivision. For the brain networks based on fMRI, the spatial coordinates of each node in three-dimensional space were used as the attributes in this study. The two-step WMM method was applied to rs-fMRI data to detect hierarchical community structures of brain networks. We compared the performance of WMM with robust MM and MM with respect to synthetic networks with planted community structures. The results demonstrated that WMM achieved better accuracy of community partitioning than MM and robust MM and better stability than MM for the synthetic networks. Moreover, the results of rs-fMRI data showed that two-step WMM showed advantages over WMM in detecting the hierarchical community structures and was more insensitive to the sparsity of the rs-fMRI networks than WMM.

The rest of the paper is organized as follows. In the theory Section, we give an overview of MM method and describe the proposed WMM and two-step WMM in detail. In materials and methods Section, the experimental design and the data processing of the simulated and real fMRI experiments are presented. The results of the experiments are presented in the results Section. In the discussion Section, we discuss and interpret the results in detail.

## Theory

### Modularity maximization (MM)

The MM method takes the adjacency matrix of the network as input and outputs the community membership of each node by maximizing the modularity function proposed in [23]. Intuitively, the modularity function compares the observed pattern of connections in a network against the pattern that would be expected under an appropriate null network. The expression of modularity function can be written as

$$\begin{array}{c} Q=\sum_{ij}[A_{ij}-P_{ij}]\delta (\sigma_{i},\sigma_{j}) \end{array}$$ (1)

In this expression, the Kronecker delta function *δ*(*σ*_*i*_, *σ*_*j*_) = 1 if *σ*_*i*_ = *σ*_*j*_ and 0 otherwise, where *σ*_*i*_ and *σ*_*j*_ are the community labels of nodes *i* and *j*. *A*_*ij*_ is the observed weight of the connection between nodes *i* and *j*, while *P*_*ij*_ is the anticipated weight of the connection between the corresponding two nodes under a specified null network. A popular choice of null network for networks with positive edge weights is the Newman-Girvan (NG) null network [23], whose adjacency matrix elements can be written as $P_{ij}\;=\;\frac{k_{i}k_{j}}{2U}$, where $k_{i}=\sum_{j=1}^{N}A_{ij}$ is the observed node strength and 2*U* is the sum of all edge weights of the network. We usually call the matrix *B* = *A* − *P* the “modularity matrix”.

For the “resolution limit” drawback mentioned in [35], Fortunato and Barthelemy introduced a tunable parameter, *γ*, to uncover communities of different sizes. Thus, the modularity function can be updated as

$$\begin{array}{c} Q=\sum_{ij}[A_{ij}-\gamma P_{ij}]\delta (\sigma_{i},\sigma_{j}) \end{array}$$ (2)

In this study, we optimized the modularity quality function using a Louvain-like locally greedy algorithm [12].

It should be noted that the modularity function includes a number of local maxima that exponentially grow with system size [36]. The local maxima are very close to the global maximum, although the corresponding partitions may be topologically quite different from each other. Therefore, the random initializations can cause the optimization algorithm to easily fall into different local maxima and obtain different partitions, which ultimately leads to the instability of MM. Different MM algorithms generally perform well on networks with strong modular structures and often succeed in finding high-modularity partitions in practice [37].

The modularity function in Eq (2) mainly depends on the adjacency matrix *A* that determines the structure of a graph. The basic idea of WMM is to enlarge the differences between the edges with high probability and the edges with low probability in the same community to form a clearer cluster structure by filtering the adjacency matrix. A weight matrix $W\in R^{N\times N}$ is defined, where *W*_*ij*_ is the probability of node *i* and node *j* being assigned to the same community across all *M* partitions. We determine the Hadamard product (⊗) through elementwise multiplication on the adjacency matrix *A* and the filter *W*. The modularity function of WMM is changed to Eq (3) by weighting the adjacency matrix *A*

$$\begin{array}{c} Q=\sum_{ij}[{W_{ij}⊗A}_{ij}-\gamma P_{ij}^{\prime }]\delta (\sigma_{i},\sigma_{j}) \end{array}$$ (3)

where $P_{ij}^{\prime }$ is the corresponding null network of the weighted adjacency matrix *W* ⊗ *A*.

Because the weight matrix *W* is obtained through MM, the weight matrix also exhibits some instability due to the instability of MM. To further improve the stability of the weight matrix, the WMM constructs *K* weight matrices *W*_*k*_(*k* = 1, 2, ⋯, *K*), runs the WMM algorithm *K* times by applying the *K* weight matrices on the modularity function, and obtains the final weight matrix *W* based on the *K* partitions. The detailed construction of *W* and the main procedure of WMM are described as follows. And the pseudocode of the WMM method is shown in Algorithm 1.

First, we performed the MM method *L* times on the adjacency matrix *A* of network *G* and obtained a “partition pool” containing *L* original partitions (line 1–4). For each partition in the “partition pool”, we constructed a nodal association matrix *T* whose entry *T*_*ij*_ = 1 if nodes *i* and *j* have been assigned to the same community and *T*_*ij*_ = 0 otherwise (line 3). Second, *M* partitions are randomly selected from the “partition pool” *K* times to construct *K* weight matrices (line 5–11). For the *i*th selection, the weight matrix *W*_*i*_ can be obtained by averaging the *M* association matrices corresponding to *M* selected partitions (line 7), and the weighted adjacency matrix $A_{i}^{\prime }$ is obtained by determining the Hadamard product based on the adjacency matrix *A* and the weight matrix *W*_*i*_ (line 8). Thus, a total of *K* weighted adjacency matrices are obtained. Third, the final weight matrix *W*′ is constructed by running MM on the *K* weighted adjacency matrices separately and averaging the corresponding *K* association matrices (line 12). Finally, MM is applied to the final weighted adjacency matrix resulting from the Hadamard product of the adjacency matrix *A* and the final weight matrix *W*′ (line 13–14).

**Algorithm 1** WMM

**Require**: Adjacency matrix *A*, partitioning pool size *L*, the number of selected partitionings *M*, selection times *K*

**Ensure**: Final partitioning *S* of the network

1: **for** *k* = 1 to *L* **do**

2:  Apply MM on *A* and obtain partitioning *S*_*k*_

3:  Construct association matrix *T*^(*k*)^ from *S*_*k*_.

4: **end for**

5: **for** *i* = 1 to *K* **do**

6:  Select *M* association matrices from the partition pool randomly

7:  $W_{i}⇐\frac{1}{M}\sum_{m=1}^{M}T^{\left(m\right)}$

8:  $A_{i}^{\prime }⇐A⊗W_{i}$

9:  Apply MM to $A_{i}^{\prime }$ and obtain partitioning $S_{i}^{\prime }$

10:  Construct association matrix *T*′^(*i*)^ from $S_{i}^{\prime }$

11: **end for**

12: $W^{\prime }⇐\frac{1}{K}\sum_{i=1}^{K}T^{\left(i\right)}$

13: *A*′′ ⇐ *A* ⊗ *W*′

14: Apply MM on *A*′′ and obtain the final partitioning *S*

### Two-step weight modularity maximization (two-step WMM)

MM is unable to detect hierarchical community structures [31] in networks. The proposed two-step WMM that can avoid such the limit of MM defines the partition criterion by taking advantage of the spatial information of fMRI data. It has been suggested that the spatial layout of neurons or brain regions is economically arranged to minimize energy costs [38]. Specifically, spatially close regions are more likely to be responsible for the same cognitive function and in the same community than spatially remote regions. Therefore, we assume that a community should be further divided if the spatial distance between brain regions within the community was significantly reduced after subdivision.

To compute the spatial distance within a community, a distance matrix *D* whose element *D*_*ij*_ is the Euclidean distance between nodes (brain regions) *i* and *j* is defined. The diagonal values of the distance matrix are set to zero. The spatial distance (*d*) of a community is defined as the mean of all elements in *D* with *N* nodes:

$$\begin{array}{c} d=\frac{1}{N^{2}}\sum_{i=1}^{N}\sum_{j=1}^{N}D_{ij} \end{array}$$ (4)

The procedure of two-step WMM includes two WMM steps, which is summarized in the pseudocode of Algorithm 2. In the first step, WMM is applied on a network to obtain the community partition (line 1). In the second step, each community obtained in the first step is further divided into subcommunities by WMM (line 2–14). After subdivision in the second step, the pre- and postsubdivision spatial distances of each community are calculated (line 3 and 5). The presubdivision distance (*d*_*i*_) of community *i* can be obtained by Eq (4). Suppose community *i* is further divided into *k* subcommunities. The spatial distance (*d*) of each subcommunity is calculated by Eq (4). The postsubdivision spatial distance ($d_{i}^{\prime }$) of community *i* is calculated by averaging *d* across all the *k* subcommunities. For each community, a permutation test is conducted to test the spatial distance differences between presubdivision and postsubdivision (line 6–9). Specifically, the community assignments of all nodes are randomly shuffled *T* = 10,000 times with the community size and community number fixed according to the partition (line 7). The 10,000 shuffled spatial distances are calculated (line 8) and ranked in ascending order (line 10). If $d_{i}^{\prime }$ is smaller than *d*_*i*_ and the true distance $d_{i}^{\prime }$ is smaller than the 5% (*p* value) smallest value of sorted distances, the further division of community *i* is accepted; otherwise, it is rejected (line 11–13).

**Algorithm 2** two-step WMM

**Require**: Adjacency matrix *A*, the number of random partitions *T*, significant level *α*

**Ensure**: Final partitioning *S* of the network

1: Apply WMM to *A* and obtain a partitioning *S* containing *k* communities *C*_1_, *C*_2_, ⋯, *C*_*k*_

2: **for** *i* = 1 to *k* **do**

3:  Calculate *d*_*before*_ of *C*_*i*_

4:  Apply WMM to *C*_*i*_ and obtain a partitioning *S*_*i*_ containing *m* sub-communities

5:  Calculate the mean distance, *d*_*after*_, of the sub-communities

6:  **for** *j*=1 to *T* **do**

7:   Randomly shuffle the node labels of *S*_*i*_ to obtain a random partition $S_{i}^{\prime }$

8:   Recalculate the mean distance of the shuffled sub-communities, *d*_*j*_

9:  **end for**

10:  Sort {*d*_1_, *d*_2_, ⋯, *d*_*T*_} in ascending order and get the sorted serial $d_{1}^{\prime },d_{2}^{\prime },\cdots ,d_{T}^{\prime }$

11:  **if** *d*_*after*_ < *d*_*before*_ and $d<d_{T\times \alpha }^{\prime }$ **then**

12:   Retain the labels of nodes in community *C*_*i*_

13:  **end if**

14: **end for**

## Materials and methods

In this section, we performed experiments on both simulated data and real rs-fMRI data. Specifically, we compared the performances of MM, robust MM and WMM based on the simulated networks without node attributes and compared the performances of WMM and two-step WMM based on the simulated networks with node attributes. Moreover, we further compared the WMM and two-step WMM based on the rs-fMRI data to demonstrate the advantage of two-step WMM in detecting the hierarchical communities.

The MM code was downloaded from the website (https://github.com/GenLouvain/GenLouvain) [12]. The MATLAB codes of robust MM and WMM were written based on the MM code. For robust MM, the size of the “partition pool” was set to 100 to remain the same as the value in [30]. For robust MM and two-step WMM, the size of the “partition pool” was set to 100, and both the number of partitions selected from the “partition pool” *M* and selection iterations *K* were set to 50. The parameter *γ* was set to 1 in WMM, robust MM and MM.

### Simulated data experiment

#### Benchmark network generation

We generated undirected weighted networks with planted nonoverlapping community structures using the C++ based software package [39] (https://www.santofortunato.net/resources).

Several parameters need to be specified to generate a network. These parameters include network size *N*, average node degree 〈*k*〉, max node degree *max*_*k*_, minimum community size *c*_*min*_, maximum community size *c*_*max*_, topological mixing coefficient *μ*_*t*_, strength mixing coefficient *μ*_*w*_, the exponent parameters, *τ*_1_ and *τ*_2_, of power law distributions that the node degrees and community sizes obey, and the exponent parameter, *β*, of the power law relation between node strengths and degrees. For a detailed interpretation of the above parameters, refer to [39]. Table 1 shows the parameter values used in the simulated experiments. Each row in Table 1 corresponds to networks with a specific *N*, 〈*k*〉, *max*_*k*_, *τ*_1_, *τ*_2_, *β* and varied *μ*_*t*_(*μ*_*w*_). Note that the convergence of network generation may not be reached when the network size is small due to the strong constraint of parameters. Thus, the maximum value of the topological and strength mixing coefficients (*μ*_*w*_ and *μ*_*w*_) was set to 0.7 rather than 0.8 for the small network size (*N*=50). Thus, there were a total of 28 parameter combinations (6 for *N* = 50, 7 for *N* = 100, and 5 for *N* = 300, 500 and 1,000, respectively) in Table 1. For each parameter combination, 50 networks were generated. Therefore, a total of 1,400 (28 × 50) networks were generated in the simulated experiments.

**Table 1:** The detailed parameters of all networks used in the simulated experiments. Note that the parameters *c*_*min*_ and *c*_*max*_ were not set, and their values were automatically chosen close to the degree sequence extremes instead.

*N*	〈*k*〉	*max*_*k*_	*μ*_*t*_ = *μ*_*ω*_	*τ*_1_	*τ*_2_	*β*
50	3	9	0.2–0.7, step 0.1	2	1	1.5
100	5	25	0.2–0.8, step 0.1			
300	15	75	0.4–0.8, step 0.1			
500	25	125	0.4–0.8, step 0.1			
1,000	50	250	0.4–0.8, step 0.1			

The topological and strength mixing parameters, *μ*_*t*_ and *μ*_*w*_, denoted the average fraction of intercommunity degree and strength, respectively [39]. Thus, these parameters can represent network noise levels. The planted community structure with higher mix parameters contains a higher noise level and is more difficult to uncover. We used the mixing parameter *μ* to represent the topological and strength mixing parameters for convenience because *μ*_*t*_ and *μ*_*w*_ were set the same in the study.

#### Partition accuracy comparison of WMM, robust MM and MM

To quantitatively assess the similarity of the two community partitions from the same network, normalized mutual information (NMI) [40] was used. Given two community partitions (*X* and *Y*) of a network with *N* nodes, the NMI is defined as

$$\begin{array}{c} NMI(X,Y)=\frac{2MI(X,Y)}{H(X)+H(Y)} \end{array}$$ (5)

where *H*(*X*) denotes the entropy of *X*, and *MI*(*X*, *Y*) denotes the mutual information of *X* and *Y*. The NMI ranges from 0 to 1, where 0 indicates that the two partitions are completely different and 1 indicates that the two partitions are identical.

For each network, the WMM, robust MM and MM methods were run 20 times, and 20 community partitions were obtained from each method. The NMI between each partition and the ground truth partition was calculated. Each network’s NMI was obtained by averaging the NMI values across the 20 partitions obtained by WMM/robust MM/MM. The NMI of each parameter combination was obtained by averaging the networks’ NMI values across 50 networks.

Nonparametric Friedman tests were performed to test the NMI differences among the three methods for each parameter combination. If the Friedman test showed significant differences, Dunn’s post hoc tests and Bonferroni correction were carried out to assess significant differences in NMI between pairwise comparisons among the three methods. All statistical tests were performed using SPSS 25.0 software (https://www.ibm.com/products/spss-statistics).

#### Stability comparison of WMM, robust MM and MM

**Fig 1:**
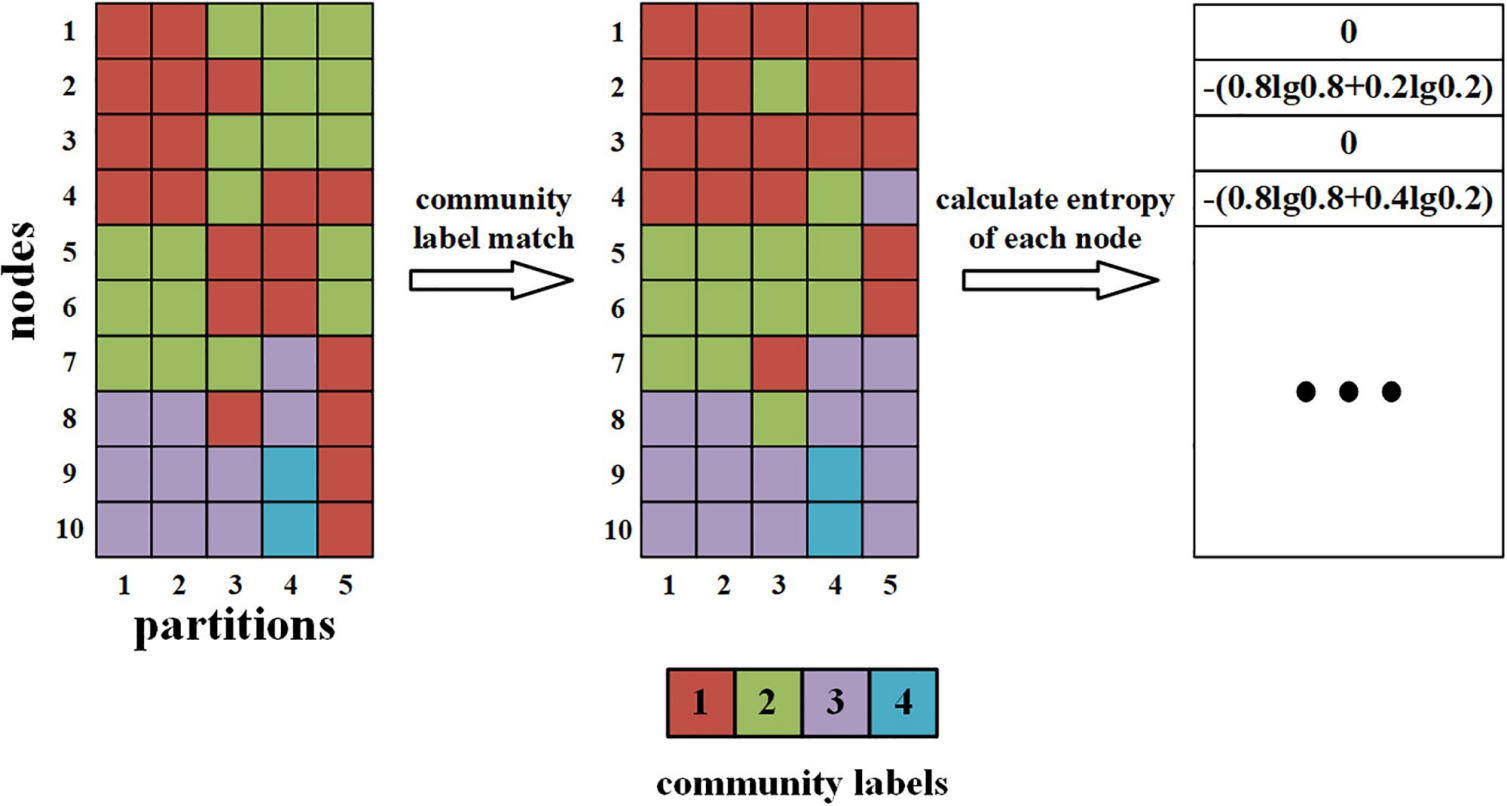
The schematic of the calculation of average node entropy for 5 partitions of a network with 10 nodes.

The NMI can effectively quantify the accuracy of community partitioning. However, NMI cannot be applied to stability evaluation because it only takes into account the overlap and ignores the nonoverlapping parts of two partitions. In this study, we proposed a measure of average node entropy to evaluate the partition stability. The core idea is using entropy to quantify the variability of the nodes’ community labels across multiple partitions because entropy can measure a state’s disorder, randomness, or uncertainty. First, we selected a partition as the “reference partition” whose community labels were used as the reference to unify the community labels of other partitions. For each partition, community label matching was performed to ensure that the community labels matched the reference partition by calculating the overlap size between the communities from the two partitions (see Fig 1). Second, we calculated the entropy of each node’s community labels that were assigned by all partitions to measure the variability of community labels across all partitions. Finally, we averaged the entropy of all nodes as the network average node entropy value to evaluate the partition stability of each method.

Because the average node entropy values may vary slightly with different selections of the “reference partition”, each partition was selected as the “reference partition” to calculate average node entropy, and the mean average node entropy was obtained by averaging all average node entropy values across all “reference partitions”. The procedure for calculating the index is shown in Fig 1.

The average node entropy of each network was calculated by using the 20 partitions that were obtained by WMM, robust MM and MM. For each parameter combination, the stability index was obtained by averaging the average node entropy values across 20 networks. Nonparametric Friedman tests were performed to test the differences in stability among the three methods under each specific parameter combination. The test procedures were the same as those in the accuracy comparison.

#### Accuracy and stability comparison of WMM and two-step WMM

We generated undirected unweighted networks with node attributes and planted nonoverlapping community structures and using the Python package X-Mark [41] (https://github.com/dsalvaz/XMark). Considering that X-Mark is an extension of the package used to generate networks without node attributes, the parameters of networks with node attributes are set similar to those of networks without node attributes. The network size *N* was set to 300. The average node degree 〈*k*〉 was set to 15. The maximum of node degree *max*_*k*_ was set to 75. The minimum community size *c*_*min*_, maximum community size *c*_*max*_ were automatically chosen close to the degree sequence extremes. The power-law exponents for node degree sequence and community size sequence, *τ*_1_ and *τ*_2_ were set to 2 and 1.5, separately. The number of continuous node attributes *m*_*cont*_ was set to 3. The mixing parameter *μ* varied from 0.4 to 0.6 with the increase of 0.1. The standard deviation of node attributes *σ* varied from 0.2 to 0.8 with the increase of 0.1. For each parameter combination of *μ* and *σ*, 20 networks were generated.

For each network, the WMM and two-step WMM methods were run 20 times, and 20 community partitions were obtained from each method. The NMI between each partition and the ground truth partition was calculated. Each network’s NMI was obtained by averaging the NMI values across the 20 partitions obtained by each method. The NMI of each parameter combination was obtained by averaging the networks’ NMI values across 20 networks. The average node entropy value of each network was calculated by using the 20 partitions that were obtained by WMM and two-step WMM. For each parameter combination, the stability index was obtained by averaging the average node entropy values across 20 networks.

Nonparametric Wilcoxon signed-rank tests were performed to test the differences in NMI and average node entropy between WMM and two-step WMM for each for each parameter combination.

### Resting state fMRI data experiment

The two-step WMM method was applied to real rs-fMRI data to investigate the feasibility and performance of two-step WMM in community detection. Because the simulated experiment demonstrated that WMM showed better performance than robust MM and MM, the partition performance of two-step WMM was further compared with WMM in the rs-fMRI experiment.

#### Subjects

The rs-fMRI dataset used in this study was from the 1200 Subjects release (S1200 release) (https://www.humanconnectome.org/study/hcp-young-adult/document/1200-subjects-data-release) in the WU-Minn Human Connectome Project (HCP) [42]. Specifically, the “100 Unrelated Subjects” (*N* = 100) subset of the S1200 release without family structure issues was used in this study. The HCP scanning protocol was approved by the local Institutional Review Board at Washington University in St. Louis, and informed written consent from each subject was obtained for data collection. The “100 Unrelated Subjects” dataset is available on the data management platform ConnectomeDB (https://db.humanconnectome.org). The study was approved by the Institutional Review Board (IRB) of the State Key Laboratory of Cognitive Neuroscience and Learning in Beijing Normal University (approval number: IRB_A_0032_2020001).

#### Data acquisition

All subjects were scanned on a customized 32-channel Siemens 3T “Connectome Skyra” housed at Washington University. Each subject underwent two approximately 15-minute resting-state scans, with eyes open and relaxed fixation on a projected bright cross-hair on a dark background. Each scan consisted of two runs with different phase encoding in the left-to-right (LR) direction in one run and right-to-left (RL) direction in the other. Whole-brain gradient-echo-planar imaging acquisitions were acquired with the following main parameters: time repetition = 720 ms, time echo = 33.1 ms, flip angle = 52°, field of view = 208×180 mm^2^, matrix = 104×90, slice thickness = 2 mm, number of slices = 72, voxel size = 2×2×2 mm^3^, multiband factor = 8. For more information about data acquisition, please refer to [43]. In this study, the rs-fMRI data of the “LR” encoded run in the first scan (i.e., REST1) were used.

#### Preprocessing

The ICA-FIX rs-fMRI data of the HCP that produced the “minimal preprocessing pipelines” [44] and ICA-FIX [45, 46] were used in the study. The minimal preprocessing pipelines included structural and functional pipelines, and ICA-FIX was used as an automatic noise detection algorithm for removing spatial and temporal artifacts such as head motion, physiological noise from fMRI data. Finally, temporal bandpass filtering (0.01–0.1 Hz) was performed using DPARSF [47] (http://rfmri.org/dpabi). The preprocessed fMRI data were used for brain network construction and further analysis.

#### Construction of the brain network

In this study, we utilized a cortical-based parcellation [48] that contained 333 regions of interest (ROIs). The time course of each cortical ROI was obtained from the preprocessed rs-fMRI data by averaging the time courses of all voxels within the ROI. We computed the Pearson correlation coefficients between the time courses of each pair of nodes and generated a 333×333 symmetric correlation matrix for each subject. Fisher’s r-to-z transformation was performed for each correlation matrix. The group-level brain network was constructed by averaging the 90 transformed correlation matrices that were randomly selected from the 100 subjects, followed by an inverted Fisher’s transformation [49]. A total of 100 group-level networks were generated by randomly selecting 90 correlation matrices 100 times. For each correlation matrix, 7 group-level brain networks with 7 densities that varied from 5% to 35% with an increase of 5% were generated. For each network density *S*, only the edges in top *S* (total 333×332/2×*S* edges) were retained to reduce the influence of weak or spurious connectivities. Notably, all negative correlations were set to zero due to their ambiguous physiological interpretations [50, 51]. Therefore, 100 group-level brain networks were obtained for each network density. The two-step WMM and WMM methods were applied to each group-level network 20 times separately to obtain 20 partitions.

#### Accuracy and stability comparison of WMM and two-step WMM

NMI was used to investigate the accuracy of the partitioning. For the real rs-fMRI data, the true community structures of functional brain networks were unknown. The predefined partition in Gordon’s study [48] was supposed to be the ground truth in this study. Thus, each partition’s NMI was calculated by comparing the detected partition and the predefined partition. Based on the predefined partition [48], the 333 ROIs were assigned to 12 subnetworks. These subnetworks were default mode network (41 ROIs), cingulo-opercular network (40 ROIs), visual network (39 ROIs), somatosensory-motor hand network (38 ROIs), dorsal attention network (32 ROIs), fronto-parietal network (24 ROIs), auditory network (24 ROIs), ventral attention network (23 ROIs), somatosensory-motor mouth network (8 ROIs), retrosplenial temporal network (8 ROIs), cingulo-parietal network (5 ROIs), and salience network (4 ROIs). Moreover, 47 nodes were unassigned, for which each subnetwork label was null.

For each group-level network, the NMIs of the 20 partitions were calculated, and the network NMI was obtained by averaging the NMIs across 20 partitions. For each network density, the mean NMI was calculated by averaging the network NMI values across the 100 group-level networks.

Average node entropy was used to quantify the stability of the partition. For each group-level network, the average node entropy values of 20 partitions were calculated in the same way as the simulated data, and the network average node entropy was obtained by averaging the 20 average node entropy values across the 20 partitions. For each network density, the mean average node entropy was calculated by averaging the network average node entropy values across the 100 group-level networks. Nonparametric Wilcoxon signed-rank tests were performed to test the differences in NMI and average node entropy between WMM and two-step WMM for each network density.

## Results

### Simulated data experiment

#### Partition accuracy comparison of WMM, robust MM and MM

**Fig 2:**
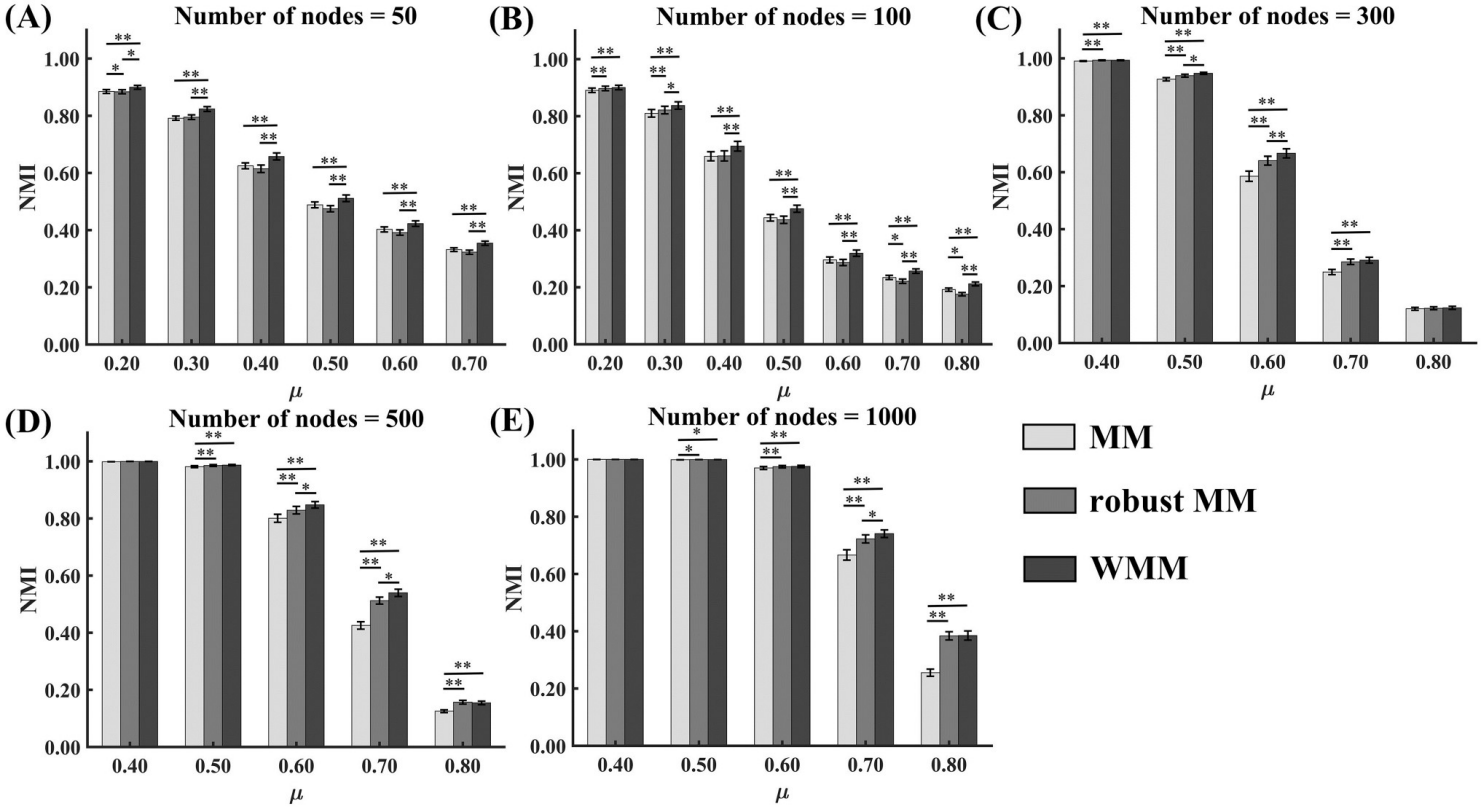
NMIs of MM, robust MM and WMM for networks without nodes attributes in the simulated data experiments. Error bars represent the standard error (* *p* < 0.05; ** *p* < 0.0005).

The NMI values of the three methods, MM, robust MM and WMM for different mixing coefficients *μ* in the cases of different network sizes are shown in Fig 2. The NMI values of all three methods decreased with the increasing mixing parameter *μ* for each fixed network size and increased with the increasing network size for each fixed mixing parameter *μ*.

Moreover, Friedman tests were carried out to compare the NMI values of the three methods. Among the three methods, WMM showed the highest NMI values in almost all cases. When the network size is small (*N* = 50 or 100), the Friedman tests showed significant NMI differences among the three methods for all noise levels. The Dunn-Bonferroni post hoc tests revealed that WMM produced significantly higher NMI values than both robust MM and MM (*p* < 0.05) in almost all cases. The post hoc tests showed that NMI values of robust MM were significantly higher than MM in some cases (*μ* = 0.2 for *N* =50 and *μ* = 0.2, 0.3, 0.7 and 0.8 for *N* = 100). When the network is large (*N* = 300, 500 or 1,000), the Friedman tests showed significant NMI differences among the three methods for medium noise level (*μ* = 0.5, 0.6 or 0.7). The Dunn-Bonferroni post hoc tests revealed that both WMM and robust MM produced significantly higher partition accuracy than MM (*p* < 0.05) in almost all cases. The post hoc tests showed that the partition accuracies of WMM were significantly higher than robust MM in some cases (*μ* = 0.5 and 0.6 for *N*=300, *μ* = 0.6 and 0.7 for *N*=500 and *μ* = 0.7 for *N* = 1000). The statistical values and *p* values of the tests are shown in S1 Table.

#### Stability comparison of WMM, robust MM and MM

**Fig 3:**
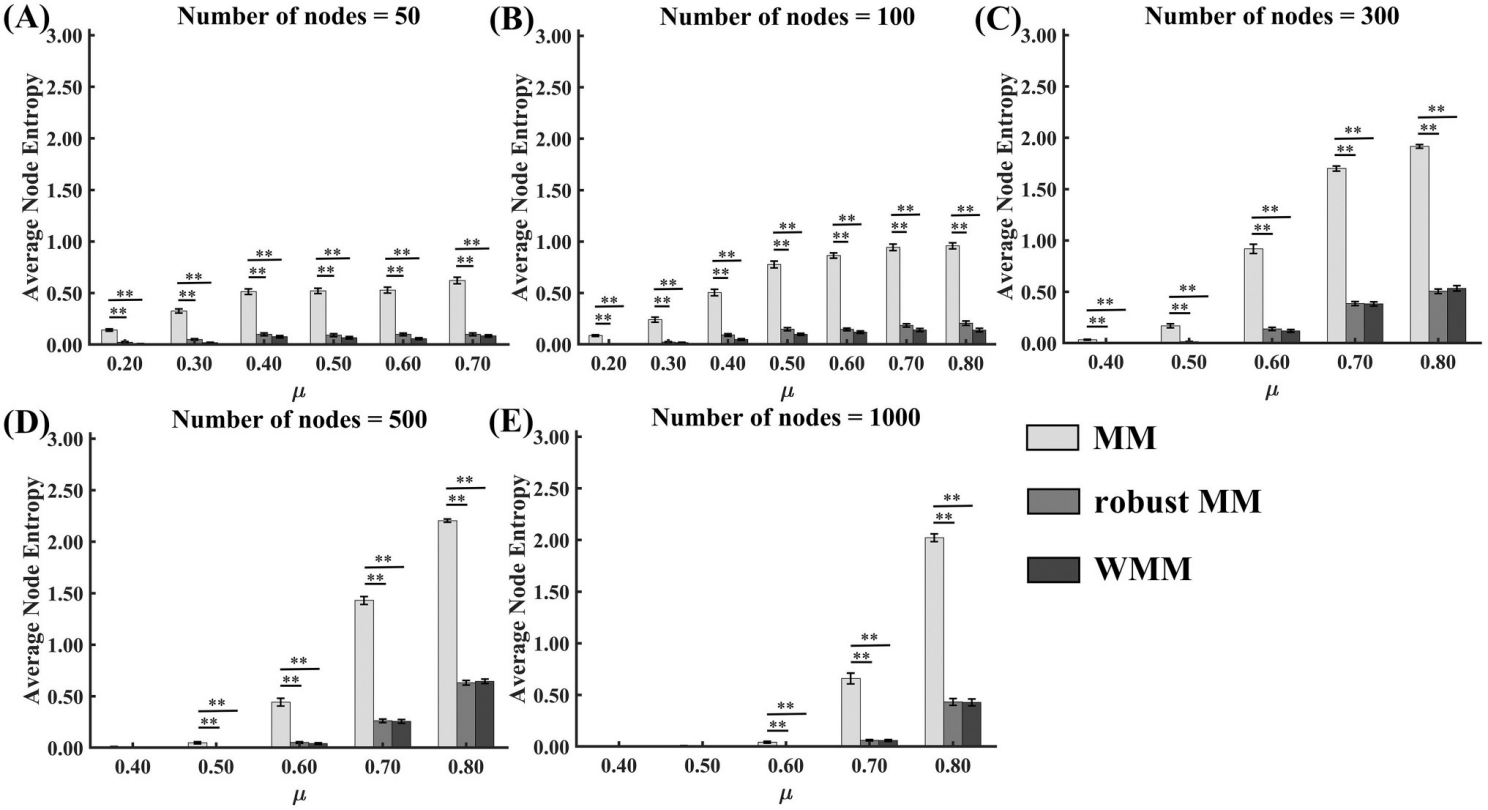
Average node entropy values of MM, robust MM and WMM for networks without nodes attributes in the simulated data experiments. Error bars represent the standard error (* *p* < 0.05; ** *p* < 0.0005).

The average node entropy values of the three methods, MM, robust MM and WMM for different mixing coefficients *μ* in the cases of different network sizes are shown in Fig 3. The average node entropy values of all three methods increased with the increasing mixing parameter *μ* for each fixed network size and increased with the increasing network size for each fixed mixing parameter *μ*. Moreover, Friedman tests were carried out to compare the average node entropy values for the three methods. Friedman tests showed significant average node entropy differences among the three methods for almost all noise levels and network sizes. The Dunn-Bonferroni post hoc tests revealed that MM produced significantly lower stability than both robust MM and WMM (*p* < 0.05) in almost all cases. No significant average node entropy differences between WMM and robust MM were found in all cases. The statistical values and *p* values of the tests are shown in S2 Table.

#### Accuracy and stability comparison of WMM and two-step WMM

**Fig 4:**
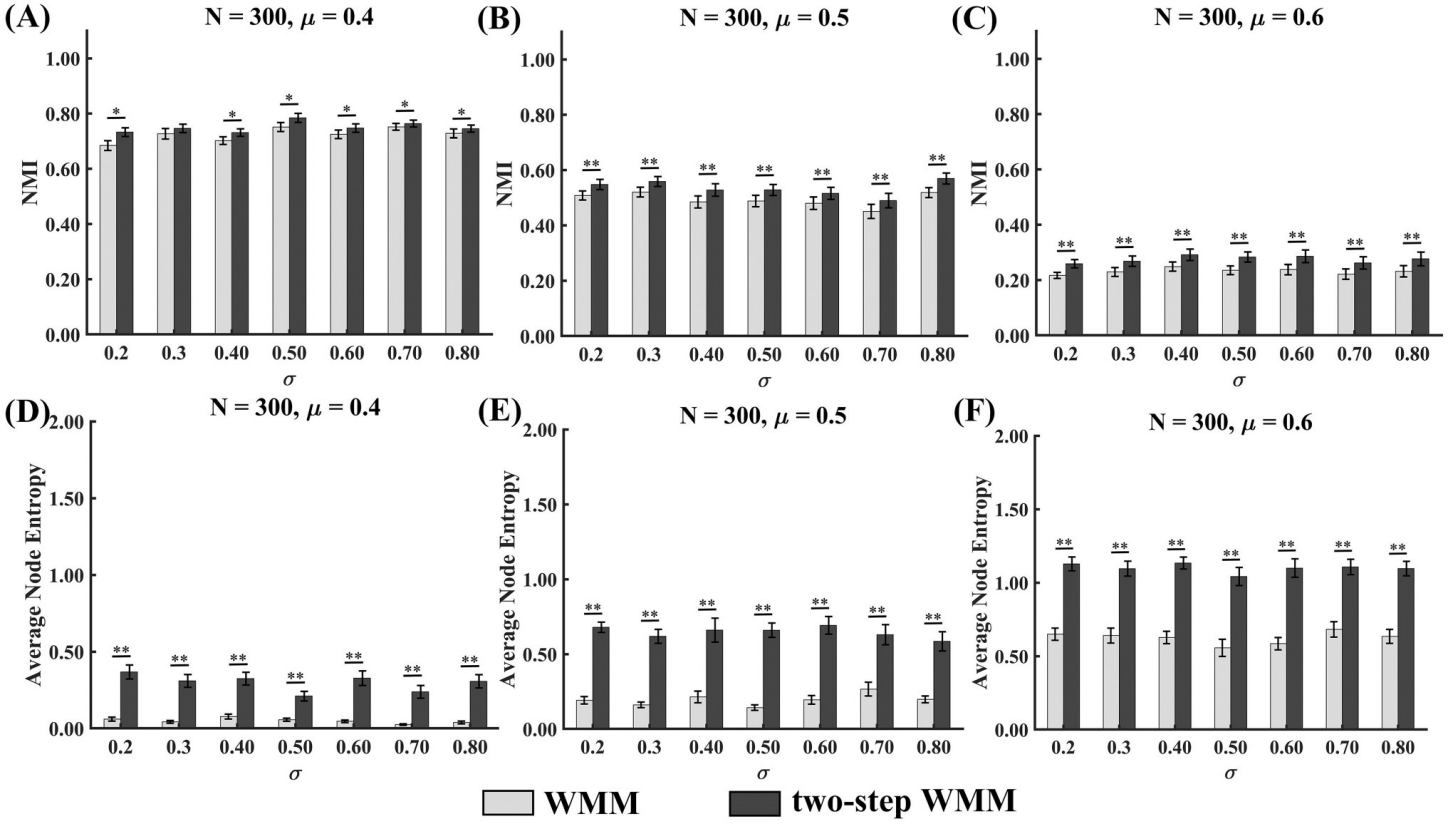
NMIs and average node entropy values of WMM and two-step WMM for networks with nodes attributes in the simulated data experiments. Error bars represent the standard error (* *p* < 0.05; ** *p* < 0.0005).

The NMI values of WMM and two-step WMM for different mixing coefficients *μ* in the cases of different standard deviations of node attributes *σ* are shown in Fig 4(A)-4(C). The NMI values of the two methods decreased with the increasing mixing parameter *μ* for each fixed standard deviation of node attributes *σ* and changed slightly with the increasing the standard deviation of node attributes *σ* for each fixed mixing parameter *μ*. Moreover, the results of Wilcoxon tests showed that two-step WMM produced significantly higher NMIs than WMM for all the mixing parameters *μ* and the standard deviations of node attributes *σ* (*p* < 0.05). The statistical values and *p* values of the tests are shown in S3 Table.

The average node entropy values of WMM and two-step WMM for different mixing coefficients *μ* in the cases of different standard deviations of node attributes *σ* are shown in Fig 4(D)-4(F). The average node entropy values of the two methods increased with the increasing mixing parameter *μ* for each fixed standard deviation of node attributes *σ* and changed slightly with the increasing the standard deviation of node attributes *σ* for each fixed mixing parameter *μ*. Moreover, the results of Wilcoxon tests showed that two-step WMM produced significantly higher average node entropy values than WMM for all mixing parameters *μ* and the standard deviations of node attributes *σ* (*p* < 0.05). The statistical values and *p* values of the tests are shown in S3 Table.

### Resting state fMRI data experiment

#### Partition accuracy comparison of WMM and two-step WMM

**Fig 5:**
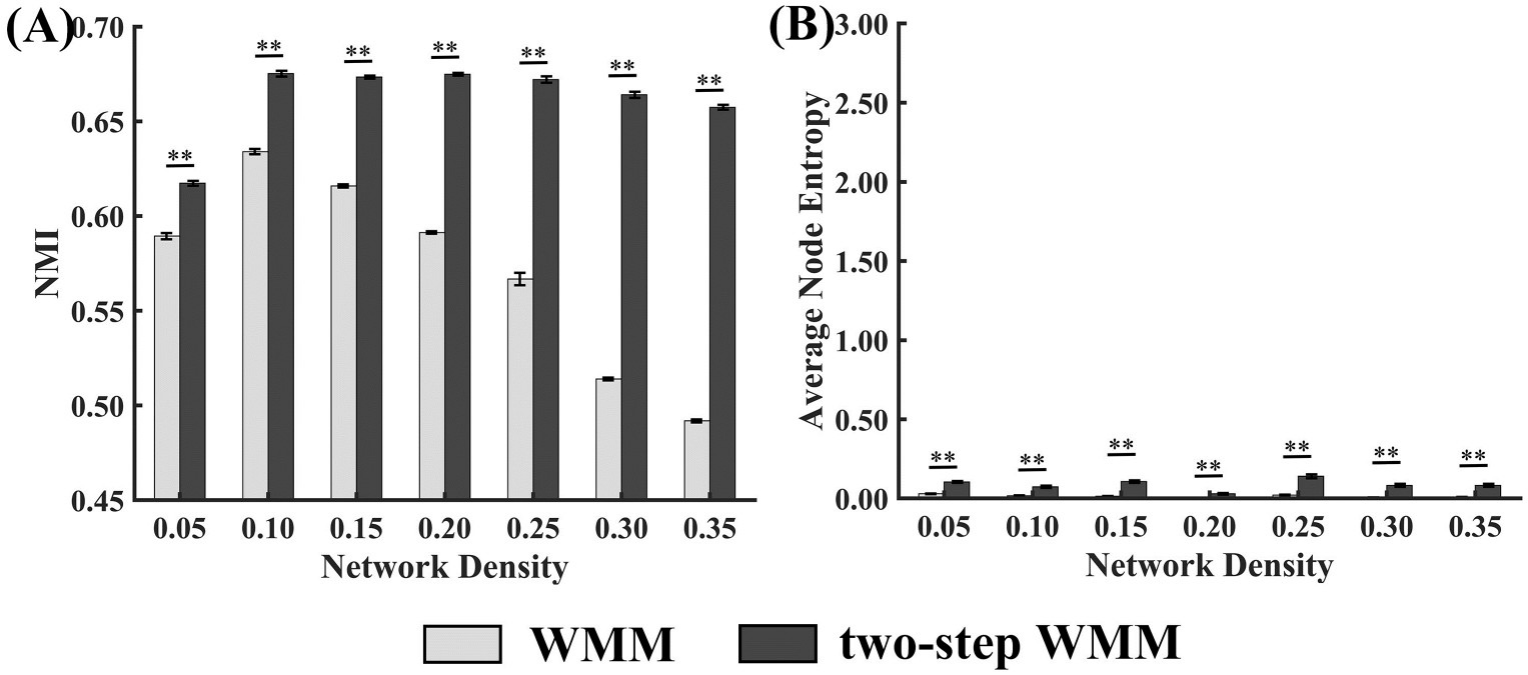
NMIs and average node entropy values of WMM and two-step WMM in rs-fMRI data experiments. Error bars represent the standard error (* *p* < 0.05; ** *p* < 0.0005).

The mean NMI values of WMM and two-step WMM for different network densities are presented in Fig 5(A). The NMI values of WMM reached the minimum and maximum when the network density was 0.35 and 0.1, respectively. For two-step WMM, the NMI values reached minimum for the network density of 0.05. When the network density varied from 0.1 to 0.35, the NMI values of two-step WMM varied slightly. Moreover, the results of Wilcoxon tests showed that two-step WMM produced significantly higher partition accuracy than WMM for all network densities. The statistical values and *p* values of the tests are shown in S4 Table.

#### Stability comparison of WMM and two-step WMM

**Fig 6:**
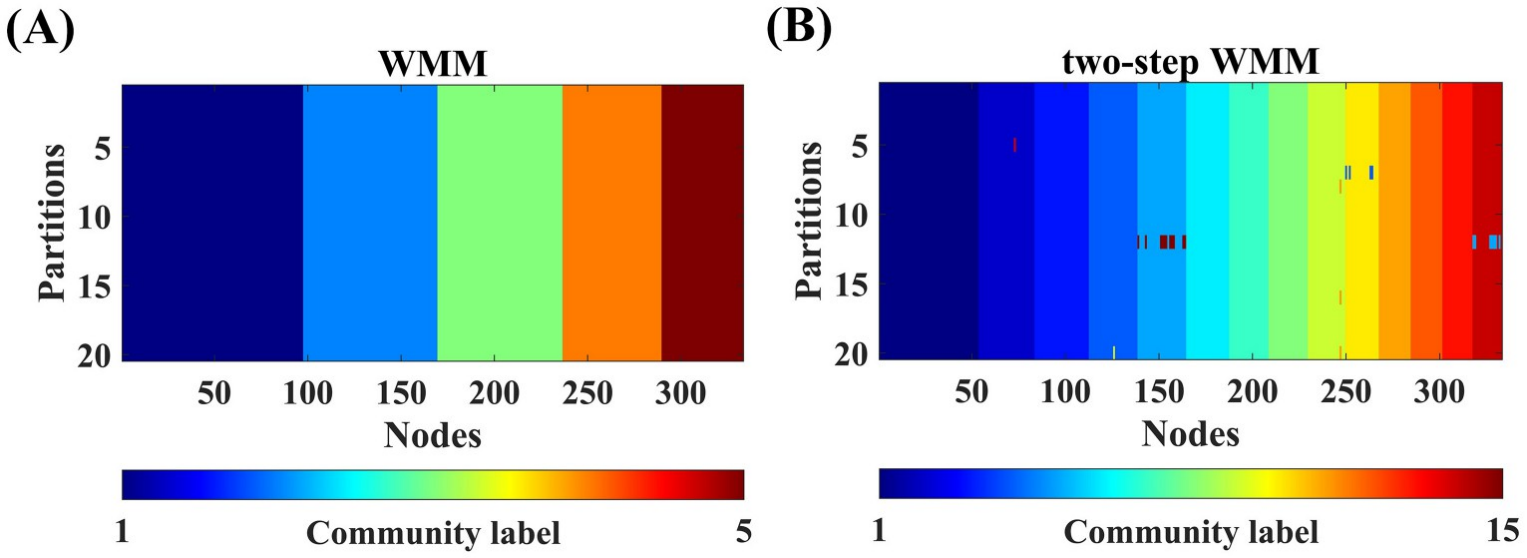
The stability visualization of WMM and two-step WMM in rs-fMRI data experiments. The network density of the group-level network used for visualization was 0.1.

The mean average node entropy values of WMM and two-step WMM for different network densities are shown in Fig 5(B). The results of Wilcoxon tests showed that two-step WMM had significantly higher average node entropy values than WMM for all network densities. The statistical values and *p* values of the tests are shown in S4 Table. Moreover, the 20 partitions of one group-level brain network (density = 0.1) are shown in Fig 6 for both WMM and two-step WMM. It can be determined that the results of two-step WMM were more stable than those of WMM across 20 partitions.

**Fig 7:**
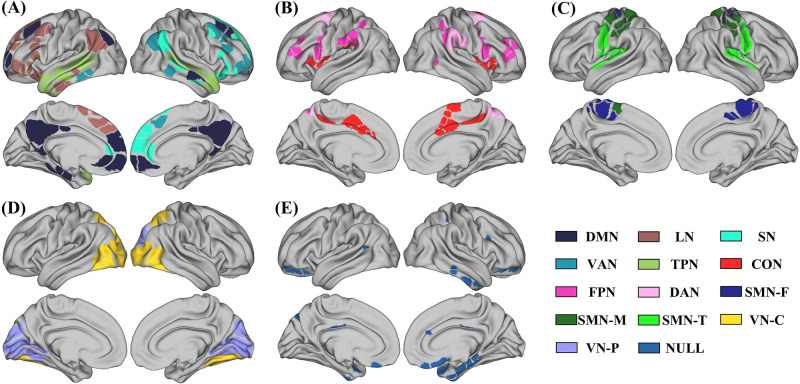
The spatial distribution of communities revealed by two-step WMM based on a representative group-level brain network. DMN: default mode network; LN: language network; SN: salience network; VAN: ventral attention network; TPN: temporal parietal network; CON: cingulo-opercular network; FPN: fronto-parietal network; DAN: dorsal attention network; SMN-F: somatosensory-motor foot network; SMN-M: somatosensory-motor mouth network; SMN-T: somatosensory-motor tongue network; VN-C: visual central network and VN-P: visual periphery network. For detailed community labels of ROIs of partitions, see S5 Table.

To compare the reasonability of community partitions obtained by WMM and two-step WMM, the spatial community distributions of the partitions of one WMM run and one two-step WMM run from Fig 6 are presented in Fig 7.

Each subfigure in Fig 7 shows the brain regions belonging to one community from the WMM’s partition. Moreover, different colors in each subfigure represent different communities that were obtained by further subdividing each WMM’s community into subcommunities through two-step WMM. Five communities were obtained by WMM (see Fig 7(A)–7(E)). The community in Fig 7(A) was the largest community that contained the default mode network, language network, salience network, ventral attention network and temporal parietal network detected by two-step WMM. The community in Fig 7(B) contained fronto-parietal network, dorsal attention network and cingulo-opercular network detected by two-step WMM. The community in Fig 7(C) contained the somatosensory-motor foot network, somatosensory-motor mouth network and somatosensory-motor tongue network detected by two-step WMM. The community in Fig 7(D) contained the visual central network and visual periphery network detected by two-step WMM. The community in Fig 7(E) only contained the NULL community detected by WMM and two-step WMM.

## Discussion

In this study, we proposed the WMM method to improve the accuracy and stability of MM by constructing two-round weight matrices to weight the adjacency matrix of a network. Based on WMM, the two-step WMM method that utilized the node attributes was further proposed to reveal the hierarchical community structures of brain networks. The simulated results demonstrated that WMM showed better accuracy of community partitioning than MM and robust MM and better stability than MM for networks without node attributes. The two-step WMM method showed better accuracy of community partitioning than WMM for networks with node attributes. Moreover, the results of rs-fMRI data showed that two-step WMM offered advantages over WMM in detecting the hierarchical communities and was more insensitive to the density of the rs-fMRI networks than WMM.

### Simulated data experiment

In the simulated experiment, the partition accuracies of MM, robust MM and WMM decreased with the increasing topological mixing parameter *μ* (*μ*_*t*_ and *μ*_*w*_) across all network scales (see Fig 2), which was consistent with a prior study [52]. The topological mixing parameter *μ* can confuse the planted community structure. The larger *μ* is, the more unclear the community structure will be. Thus, the mixing parameter *μ* indirectly reflects the noise level of networks. It is reasonable that the three methods showed reduced partition accuracy when the noise levels increased. Moreover, the three methods showed better robustness to the noise level in the case of a large network size (see Fig 2), which may suggest that the three methods had a greater advantage in detecting communities of large-scale networks [12].

Among the three methods, MM showed the worst partition accuracy in almost all cases. In contrast to robust MM and MM, WMM showed significantly better partitioning performance in most cases, especially for the small network and the large networks with medium noise levels. For WMM, the weight matrix was derived from the association matrix that was estimated by multiple MM runs and represented the probability of two nodes being in the same community. When the adjacency matrix of the network was weighted by the weight matrix, the differences between the edges with high probability belonging to the intracommunity and the edges with high probability belonging to the intercommunity were magnified. Thus, the weighted adjacency matrix should have clearer clustered structures than the original adjacency matrix, which possibly largely enhanced the partitioning performance of WMM. The robust MM method performs partitioning on the association matrix instead of the adjacency matrix. Although the association matrix included the probability of intracommunity edges, it lost the weight information of edges. Because the WMM method integrated both the probability and weight information of edges, it showed the best partitioning performance among the three methods.

When the network size was increased, the stabilities of MM, robust MM and WMM increased for the small and medium noise levels (*μ* < 0.7) (see Fig 3), which may indicate that the three methods were more stable in large networks than small networks when the noise was not very large. Better stability and robustness to noise in large networks for the three methods may imply that the modularity maximization methods were superior with respect to large networks. Among the three methods, MM showed the worst stability in all cases, while robust MM and WMM did not show significant differences in stability. The better stability of robust MM has been demonstrated by a previous study [30]. For WMM, the weighted adjacency matrix is useful in improving the stability of WMM to some extent because the NMI results of the simulated data indicated that WMM showed better robustness to noise (see Fig 2). Moreover, WMM constructed a fixed “partition pool”, and the first-round weight matrices were constructed from the partition pool. Considering the instability of the first-round weight matrices, the second-round weight matrix was constructed by applying the first-round weight matrices to the adjacency matrix for the WMM. Better stability of the second-round weight matrix further improved the stability of the WMM.

For the network with node attributes, the partition accuracies of two-step WMM were significantly higher than those of WMM in almost all cases. The higher partition accuracy of two-step WMM may suggest that the second-step structure partition of some communities by using the node attributes contributed to the improvement of partition accuracy. Previous studies demonstrated that MM may sufer from merging small communities into one large community [33], which could result in the worse partition accuracy of WMM than that of two-step WMM. However, the stabilities of two-step WMM were significantly worse than those of WMM in all cases. The network scale of the second step of two-step WMM was reduced largely, ranging from 50 to 100. The simulated results of networks without node attributes demonstrated that the stability of small-scale networks (*N* = 50 and 100) was worse than that of large-scale networks (*N* = 300) for the small and medium noise levels (*μ* < 0.6). Therefore, two-step WMM showed lower stability compared to WMM.

### Resting state fMRI data experiment

The results of the rs-fMRI data experiment showed that the partition accuracies of two-step WMM were significantly higher than those of WMM across all network densities. When the network density increased from 0.1 to 0.35, the partition accuracy of two-step WMM changed slightly, while the partition accuracy of WMM decreased sharply (see Fig 5(A)). These results may suggest that the community partitioning of two-step WMM was closer to the previous template and that two-step WMM was more robust to the network density than WMM. The increase in spurious edges in the network with the increasing density could confuse the community structure of the network, which could largely decrease the partition performance of the WMM. For two-step WMM, the second WMM step was performed on the communities detected by the first WMM step. Each first-step community should contain fewer nodes and spurious edges than the whole brain network due to the filtering of the first WMM step. Therefore, the increasing density did not degenerate the performance of two-step WMM. Moreover, the combination of functional and structural information is helpful for two-step WMM to reveal the hierarchical community structure (see Fig 7). It is worth noting that the partiion accuracy of two-step WMM with a network density of 0.05 was much lower than that of other densities. The reason for this was that too few edges could result in the occurrence of many isolated nodes when the density was too low.

The stability of WMM and two-step WMM were high across all network densities; however, WMM showed better stability than two-step WMM according to the results of nonparametric Wilcoxon tests (see Fig 5(B)). The instability of two-step WMM method mainly comes from obtaining subcommunities from communities obtained from WMM (refer to line 4, Algorithm 2). The network scale of the second step of two-step WMM was largely reduced and ranged from 50 to 100. The simulated experiments demonstrated that the stability of small-scale networks (*N* = 50 and 100) was worse than that of large-scale networks (*N* = 300) for the small and medium noise levels (*μ* < 0.6). Because the noise of preprpcessed rs-fMRI data was not very large, the larger instability of two-step WMM could be attributed to the reduced network nodes of the second step of two-step WMM.

The communities obtained by two-step WMM corresponded well with the well-known resting state functional networks of the human brain [16, 34]. WMM detected five communities while two-step WMM further divided the four first-level communities into 13 second-level communities. In contrast to WMM, two-step WMM can successfully reveal the hierarchical community structures of brain network by combining the adjacent matrix and node attributes.

The first WMM community in Fig 7(A) was subdivided into the default mode network, language network, salience network, ventral attention network and temporal parietal network by two-step WMM. The default mode network included the “hub” regions containing the posterior cingulate cortex and anterior medial prefrontal cortex, the “medial temporal lobe subsystem” containing the ventral medial prefrontal cortex, posterior inferior parietal lobule, parahippocampal cortex, and hippocampus formation, and some regions in the dorsal medial prefrontal cortex subsystem, which was consistent with prior studies [53, 54]. The language network, which is left-lateralized, mainly contained language-sensitive regions such as the opercular/triangular part of the inferior frontal gyrus, middle frontal gyrus, and anterior temporal regions [55]. The salience network included the anterior insula, dorsal anterior cingulate cortex and superior frontal gyrus, which are associated with self-awareness. Self-awareness is an important function of the salience network [56]. The core regions in the ventral attention network are the temporal-parietal junction and middle frontal gyrus that is a component of the ventral frontal cortex [57, 58]. One of the most important properties of the ventral attention network was lateralization to the right hemisphere, which was consistent with previous studies [57, 58]. The temporal parietal network includes regions mainly located in the superior and middle temporal gyrus [34].

The second WMM community in Fig 7(B) was further subdivided into fronto-parietal network, dorsal attention network and cingulo-opercular network by two-step WMM. The fronto-parietal network consists of the anterior cingulate cortex, dorsolateral prefrontal cortex and inferior parietal lobule [59, 60]. The core regions in the dorsal attention network were the intraparietal sulcus and the junction of the precentral and superior frontal sulcus [58]. Cingulo-opercular network consists of the dorsal anterior cingulate cortex/medial superior frontal cortex and anterior insula/frontal operculum [61]. Moreover, these three subcommunities made up the “task-positive” system that is broadly activated across tasks [16, 62]. The third WMM community in Fig 7(C) was further subdivided into three subnetworks that were related to sensory and motor function. We named them somatosensory-motor foot network, somatosensory-motor mouth network and somatosensory-motor tongue network, according to their different functions and prior studies [34, 63]. A similar division was found [16]. The fourth WMM community in Fig 7(D) was subdivided into the two communities that were related to vision. We named the two subnetworks visual central network and visual periphery network according to the study by [34]. In Fig 7(E), the null community contained small communities with isolated or no more than five ROIs and was not further divided.

It should be noted that the auditory network was included in somatosensory-motor tongue network, which was similar to previous reports [34]. This observation perhaps reflected a polysynaptic circuit of functional coupling linked to speech movements and hearing one’s own voice, according to [34]. From the perspective of the method itself, the strong connectivities and spatial proximity made it difficult to separate the two parts. Moreover, due to the hard-partition property of the MM method, one ROI can only belong to one community. For example, the intraparietal sulcus is an overlap region of the fronto-parietal network and dorsal attention network [58, 59], and it belongs to the fronto-parietal network according to the results of two-step WMM (see Fig 7). In addition, the anterior insula and dorsal anterior cingulate cortex are core regions of both the dorsal attention network and salience network and are included in the dorsal attention network in the two-step WMM.

## Conclusion

In this study, we proposed the WMM method to improve the stability and accuracy of MM. Moreover, we further proposed two-step WMM method to detect the hierarchical community structures of brain networks. The simulated results demonstrated that WMM showed better accuracy of community partitioning than MM and robust MM and better stability than MM. The two-step WMM method showed better accuracy of community partitioning than WMM for synthetic networks with node attributes. The results of rs-fMRI data showed that two-step WMM has the advantage of detecting the hierarchical communities over WMM and was more insensitive to the density of the rs-fMRI networks than WMM.

## Supporting information

S1 Table The results of nonparametric tests of NMI for networks without nodes attributes in the simulated experiments. (DOCX)

S2 Table The results of nonparametric tests of average node entropy for networks without nodes attributes in the simulated experiments. (DOCX)

S3 Table The results of nonparametric tests of NMI and average node entropy for networks with nodes attributes in the rs-fMRI data experiments. (DOCX)

S4 Table The results of nonparametric tests of NMI and average node entropy in the rs-fMRI data experiments. (DOCX)

S5 Table The community labels of all ROIs of partitions obtained by WMM and two-step WMM in Fig 7. We also showed the community labels of 333 regions from Gordon’s parcellation. The correspondence between community label in two-step WMM and functional networks: 1: NULL; 2: cingulo-opercular network; 3: somatosensory-motor tongue network; 4: default mode network; 5: fronto-parietal network; 6: visual central network; 7: somatosensory-motor mouth network; 8: visual periphery network; 9: language network; 10: salience network; 11: ventral attention network; 12: somatosensory-motor foot network; 13: temporal parietal network;14: dorsal attention network.
(DOCX)
