# Supplementary material for: Improved brain community structure detection by two-step weighted modularity maximization
Source: PLoS One. 2023 Dec 8;18(12):e0295428. doi: 10.1371/journal.pone.0295428 (PMC10707683; doi:10.1371/journal.pone.0295428)
Supplement: S1 Table — (DOCX) [file pone.0295428.s001.docx]

**S1 Table. The results of nonparametric tests of NMI for networks without**

**nodes attributes in the simulated experiments.**

| $\boldsymbol{N}$ | $\boldsymbol{\mu}$ | **Friedman**  **Test** | | **Dunn-Bonferroni Post Hoc Tests** | | | | | |
| --- | --- | --- | --- | --- | --- | --- | --- | --- | --- |
|  |  |  |  | **WMM-MM** | | **WMM-robust MM** | | **robust MM-MM** | |
|  |  | **Test Statistic (**$\boldsymbol{\chi}$***^2^*)** | **Sig.** | **Std. Test Statistic (**$\boldsymbol{Z}$**)** | **Adj. Sig.** | **Test Statistic (**$\boldsymbol{Z}$**)** | **Adj. Sig.** | **Test Statistic (**$\boldsymbol{Z}$**)** | **Adj. Sig.** |
| 50 | 0.2 | 43.646 | <0.0005 | 6.150 | <0.0005 | 3.600 | 0.001 | 2.550 | 0.032 |
|  | 0.3 | 55.508 | <0.0005 | 7.050 | <0.0005 | 5.100 | <0.0005 | 1.950 | 0.154 |
|  | 0.4 | 36.985 | <0.0005 | 4.750 | <0.0005 | 5.600 | <0.0005 | 0.850 | 1.000 |
|  | 0.5 | 45.716 | <0.0005 | 4.350 | <0.0005 | 6.600 | <0.0005 | 2.250 | 0.073 |
|  | 0.6 | 56.612 | <0.0005 | 5.500 | <0.0005 | 7.100 | <0.0005 | 1.600 | 0.329 |
|  | 0.7 | 51.543 | <0.0005 | 5.450 | <0.0005 | 6.700 | <0.0005 | 1.250 | 0.634 |
| 100 | 0.2 | 73.481 | <0.0005 | 7.200 | <0.0005 | 1.200 | 0.690 | 6.000 | <0.0005 |
|  | 0.3 | 62.994 | <0.0005 | 7.550 | <0.0005 | 3.700 | 0.001 | 3.850 | <0.0005 |
|  | 0.4 | 42.426 | <0.0005 | 6.150 | <0.0005 | 4.800 | <0.0005 | 1.350 | 0.531 |
|  | 0.5 | 53.759 | <0.0005 | 5.550 | <0.0005 | 6.900 | <0.0005 | 1.350 | 0.531 |
|  | 0.6 | 59.880 | <0.0005 | 5.400 | <0.0005 | 7.500 | <0.0005 | 2.100 | 0.107 |
|  | 0.7 | 63.840 | <0.0005 | 5.400 | <0.0005 | 7.800 | <0.0005 | 2.400 | 0.049 |
|  | 0.8 | 69.160 | <0.0005 | 4.600 | <0.0005 | 8.300 | <0.0005 | 3.700 | 0.001 |
| 300 | 0.4 | 83.354 | <0.0005 | 6.350 | <0.0005 | 0.100 | 1.000 | 6.250 | <0.0005 |
|  | 0.5 | 62.088 | <0.0005 | 7.350 | <0.0005 | 2.400 | 0.049 | 4.950 | <0.0005 |
|  | 0.6 | 92.280 | <0.0005 | 9.600 | <0.0005 | 4.500 | <0.0005 | 5.100 | <0.0005 |
|  | 0.7 | 70.120 | <0.0005 | 7.700 | <0.0005 | 1.000 | 0.952 | 6.700 | <0.0005 |
|  | 0.8 | 6.040 | 0.049 | 2.300 | 0.064 | 1.900 | 0.172 | 0.400 | 1.000 |
| 500 | 0.4 | 62.000 | <0.0005 | 4.650 | <0.0005 | 0.000 | 1.000 | 4.650 | <0.0005 |
|  | 0.5 | 75.835 | <0.0005 | 6.150 | <0.0005 | 0.000 | 1.000 | 6.150 | <0.0005 |
|  | 0.6 | 80.963 | <0.0005 | 8.650 | <0.0005 | 3.200 | 0.004 | 5.450 | <0.0005 |
|  | 0.7 | 84.000 | <0.0005 | 9.000 | <0.0005 | 3.000 | 0.008 | 6.000 | <0.0005 |
|  | 0.8 | 70.120 | <0.0005 | 6.700 | <0.0005 | 1.000 | 0.952 | 7.700 | <0.0005 |
| 1000 | 0.4 | 16.000 | <0.0005 | 1.200 | 0.690 | 0.000 | 1.000 | 1.200 | 0.690 |
|  | 0.5 | 44.000 | <0.0005 | 3.300 | 0.003 | 0.000 | 1.000 | 3.300 | 0.003 |
|  | 0.6 | 60.232 | <0.0005 | 5.900 | <0.0005 | 0.700 | 1.000 | 5.200 | <0.0005 |
|  | 0.7 | 87.960 | <0.0005 | 9.300 | <0.0005 | 3.600 | 0.001 | 5.700 | <0.0005 |
|  | 0.8 | 75.000 | <0.0005 | 7.500 | <0.0005 | 0.000 | 1.000 | 7.500 | <0.0005 |
