# Supplementary material for: Improved brain community structure detection by two-step weighted modularity maximization
Source: PLoS One. 2023 Dec 8;18(12):e0295428. doi: 10.1371/journal.pone.0295428 (PMC10707683; doi:10.1371/journal.pone.0295428)
Supplement: S2 Table — (DOCX) [file pone.0295428.s002.docx]

**S2 Table. The results of nonparametric tests of average node entropy for**

**networks without nodes attributes in the simulated experiments.**

| $\boldsymbol{N}$ | $\boldsymbol{\mu}$ | **Friedman**  **Test** | | **Dunn-Bonferroni Post Hoc Tests** | | | | | |
| --- | --- | --- | --- | --- | --- | --- | --- | --- | --- |
|  |  |  |  | **WMM-MM** | | **WMM-robust MM** | | **robust MM-MM** | |
|  |  | **Test Statistic (**$\boldsymbol{\chi}$***^2^*)** | **Sig.** | **Std. Test Statistic (**$\boldsymbol{Z}$**)** | **Adj. Sig.** | **Test Statistic (**$\boldsymbol{Z}$**)** | **Adj. Sig.** | **Test Statistic (**$\boldsymbol{Z}$**)** | **Adj. Sig.** |
| 50 | 0.2 | 85.318 | <0.0005 | 7.700 | <0.0005 | 0.700 | 1.000 | 7.000 | <0.0005 |
|  | 0.3 | 83.185 | <0.0005 | 8.450 | <0.0005 | 1.900 | 0.172 | 6.550 | <0.0005 |
|  | 0.4 | 76.571 | <0.0005 | 7.600 | <0.0005 | 0.200 | 1.000 | 7.400 | <0.0005 |
|  | 0.5 | 79.340 | <0.0005 | 8.200 | <0.0005 | 1.400 | 0.485 | 6.800 | <0.0005 |
|  | 0.6 | 82.408 | <0.0005 | 8.700 | <0.0005 | 2.400 | 0.049 | 6.300 | <0.0005 |
|  | 0.7 | 76.152 | <0.0005 | 7.550 | <0.0005 | 0.100 | 1.000 | 7.450 | <0.0005 |
| 100 | 0.2 | 89.037 | <0.0005 | 7.500 | <0.0005 | 0.300 | 1.000 | 7.200 | <0.0005 |
|  | 0.3 | 83.810 | <0.0005 | 7.550 | <0.0005 | 0.100 | 1.000 | 7.450 | <0.0005 |
|  | 0.4 | 78.531 | <0.0005 | 8.200 | <0.0005 | 1.400 | 0.485 | 6.800 | <0.0005 |
|  | 0.5 | 80.693 | <0.0005 | 8.650 | <0.0005 | 2.300 | 0.064 | 6.350 | <0.0005 |
|  | 0.6 | 76.440 | <0.0005 | 8.100 | <0.0005 | 1.200 | 0.690 | 6.900 | <0.0005 |
|  | 0.7 | 76.960 | <0.0005 | 8.200 | <0.0005 | 1.400 | 0.485 | 6.800 | <0.0005 |
|  | 0.8 | 79.809 | <0.0005 | 8.550 | <0.0005 | 2.100 | 0.107 | 6.450 | <0.0005 |
| 300 | 0.4 | 83.354 | <0.0005 | 6.350 | <0.0005 | 0.100 | 1.000 | 6.250 | <0.0005 |
|  | 0.5 | 86.862 | <0.0005 | 8.450 | <0.0005 | 1.900 | 0.172 | 6.550 | <0.0005 |
|  | 0.6 | 76.000 | <0.0005 | 8.000 | <0.0005 | 1.000 | 0.952 | 7.000 | <0.0005 |
|  | 0.7 | 75.160 | <0.0005 | 7.700 | <0.0005 | 0.400 | 1.000 | 7.300 | <0.0005 |
|  | 0.8 | 75.360 | <0.0005 | 7.800 | <0.0005 | 0.600 | 1.000 | 7.200 | <0.0005 |
| 500 | 0.4 | 62.000 | <0.0005 | 4.650 | <0.0005 | 0.000 | 1.000 | 4.650 | <0.0005 |
|  | 0.5 | 83.474 | <0.0005 | 6.550 | <0.0005 | 0.200 | 1.000 | 6.350 | <0.0005 |
|  | 0.6 | 81.153 | <0.0005 | 8.150 | <0.0005 | 1.300 | 0.581 | 6.850 | <0.0005 |
|  | 0.7 | 75.360 | <0.0005 | 7.800 | <0.0005 | 0.600 | 1.000 | 7.200 | <0.0005 |
|  | 0.8 | 75.160 | <0.0005 | 7.700 | <0.0005 | 0.400 | 1.000 | 7.300 | <0.0005 |
| 1000 | 0.4 | 16.000 | <0.0005 | 1.200 | 0.690 | 0.000 | 1.000 | 1.200 | 0.690 |
|  | 0.5 | 44.000 | <0.0005 | 3.300 | 0.003 | 0.000 | 1.000 | 3.300 | 0.003 |
|  | 0.6 | 80.522 | <0.0005 | 6.600 | <0.0005 | 0.300 | 1.000 | 6.300 | <0.0005 |
|  | 0.7 | 76.440 | <0.0005 | 8.100 | <0.0005 | 1.200 | 0.690 | 6.900 | <0.0005 |
|  | 0.8 | 75.640 | <0.0005 | 7.900 | <0.0005 | 0.800 | 1.000 | 7.100 | <0.0005 |
