# Supplementary material for: Improved brain community structure detection by two-step weighted modularity maximization
Source: PLoS One. 2023 Dec 8;18(12):e0295428. doi: 10.1371/journal.pone.0295428 (PMC10707683; doi:10.1371/journal.pone.0295428)
Supplement: S3 Table — (DOCX) [file pone.0295428.s003.docx]

**S3 Table. The results of nonparametric tests of NMI and average node**

**entropy for networks with nodes attributes in the simulated experiments.**

| $\boldsymbol{\mu}$ | $\boldsymbol{\sigma}$ | **NMI** | | **Average node entropy** | |
| --- | --- | --- | --- | --- | --- |
|  |  | **Wilcoxon Signed-Rank Test** | | **Wilcoxon Signed-Rank Test** | |
|  |  | **Std. Test Statistic (**$\boldsymbol{Z}$**)** | **Sig.** | **Std. Test Statistic (**$\boldsymbol{Z}$**)** | **Sig.** |
| 0.4 | 0.2 | 3.323 | 0.001 | 3.920 | <0.0005 |
|  | 0.3 | 1.904 | 0.057 | 3.920 | <0.0005 |
|  | 0.4 | 3.248 | 0.001 | 3.696 | <0.0005 |
|  | 0.5 | 3.173 | 0.002 | 3.659 | <0.0005 |
|  | 0.6 | 2.464 | 0.014 | 3.883 | <0.0005 |
|  | 0.7 | 1.979 | 0.048 | 3.920 | <0.0005 |
|  | 0.8 | 2.352 | 0.019 | 3823 | <0.0005 |
| 0.5 | 0.2 | 3.920 | <0.0005 | 3.920 | <0.0005 |
|  | 0.3 | 3.920 | <0.0005 | 3.920 | <0.0005 |
|  | 0.4 | 3.920 | <0.0005 | 3.920 | <0.0005 |
|  | 0.5 | 3.920 | <0.0005 | 3.920 | <0.0005 |
|  | 0.6 | 3.883 | <0.0005 | 3.920 | <0.0005 |
|  | 0.7 | 3.920 | <0.0005 | 3.920 | <0.0005 |
|  | 0.8 | 3.920 | <0.0005 | 3.920 | <0.0005 |
| 0.6 | 0.2 | 3.920 | <0.0005 | 3.920 | <0.0005 |
|  | 0.3 | 3.920 | <0.0005 | 3.920 | <0.0005 |
|  | 0.4 | 3.920 | <0.0005 | 3.920 | <0.0005 |
|  | 0.5 | 3.920 | <0.0005 | 3.920 | <0.0005 |
|  | 0.6 | 3.920 | <0.0005 | 3.920 | <0.0005 |
|  | 0.7 | 3.920 | <0.0005 | 3.920 | <0.0005 |
|  | 0.8 | 3.920 | <0.0005 | 3.920 | <0.0005 |
