# Supplementary material for: Improved brain community structure detection by two-step weighted modularity maximization
Source: PLoS One. 2023 Dec 8;18(12):e0295428. doi: 10.1371/journal.pone.0295428 (PMC10707683; doi:10.1371/journal.pone.0295428)
Supplement: S4 Table — (DOCX) [file pone.0295428.s004.docx]

**S4 Table. The results of nonparametric tests of NMI and average node**

**entropy in the rs-fMRI data experiments.**

| **Network Density** | **NMI** | | **Average node entropy** | |
| --- | --- | --- | --- | --- |
|  | **Wilcoxon Signed-Rank Test** | | **Wilcoxon Signed-Rank Test** | |
|  | **Std. Test Statistic (**$\boldsymbol{Z}$**)** | **Sig.** | **Std. Test Statistic (**$\boldsymbol{Z}$**)** | **Sig.** |
| 0.05 | 7.877 | <0.0005 | 8.595 | <0.0005 |
| 0.10 | 8.682 | <0.0005 | 8.560 | <0.0005 |
| 0.15 | 8.682 | <0.0005 | 8.551 | <0.0005 |
| 0.20 | 8.682 | <0.0005 | 8.055 | <0.0005 |
| 0.25 | 8.682 | <0.0005 | 7.843 | <0.0005 |
| 0.30 | 8.682 | <0.0005 | 7.323 | <0.0005 |
| 0.35 | 8.682 | <0.0005 | 7.866 | <0.0005 |
