# Supplementary material for: Improved brain community structure detection by two-step weighted modularity maximization
Source: PLoS One. 2023 Dec 8;18(12):e0295428. doi: 10.1371/journal.pone.0295428 (PMC10707683; doi:10.1371/journal.pone.0295428)
Supplement: S5 Table — We also showed the community labels of 333 regions from Gordon’s parcellation. The correspondence between community label in two-step WMM and functional networks: 1: NULL; 2: cingulo-opercular network; 3: somatosensory-motor tongue network; 4: default mode network; 5: fronto-parietal network; 6: visual central network; 7: somatosensory-motor mouth network; 8: visual periphery network; 9: language network; 10: salience network; 11: ventral attention network; 12: somatosensory-motor foot network; 13: temporal parietal network;14: dorsal attention network. (DOCX) [file pone.0295428.s005.docx]

**S5 Table. The community labels of all ROIs of partitions obtained by**

**WMM and two-step WMM in Fig 7.** We also showed the community labels of 333

regions from Gordon’s parcellation. The correspondence between community label in

two-step WMM and functional networks: 1: NULL; 2: cingulo-opercular network; 3:

somatosensory-motor tongue network; 4: default mode network; 5: fronto-parietal

network; 6: visual central network; 7: somatosensory-motor mouth network; 8: visual

periphery network; 9: language network; 10: salience network; 11: ventral attention

network; 12: somatosensory-motor foot network; 13: temporal parietal network;14:

dorsal attention network.

| **Parcel ID** | **Hem** | **Centroid (MNI)** | | | **Community in WMM** | **Community in two-step WMM** | **Community**  **in Gordon** |
| --- | --- | --- | --- | --- | --- | --- | --- |
|  |  | X | Y | Z |  |  |  |
| 1 | L | -11.2 | -52.4 | 36.5 | 1 | 4 | Default |
| 2 | L | -18.8 | -48.7 | 65 | 3 | 7 | SMhand |
| 3 | L | -51.8 | -7.8 | 38.5 | 3 | 3 | SMmouth |
| 4 | L | -11.7 | 26.7 | 57 | 1 | 9 | Default |
| 5 | L | -18.4 | -85.5 | 21.6 | 5 | 6 | Visual |
| 6 | L | -47.2 | -58 | 30.8 | 1 | 9 | Default |
| 7 | L | -38.1 | 48.8 | 10.5 | 2 | 5 | FrontoParietal |
| 8 | L | -16.8 | -60.1 | -5.4 | 5 | 8 | Visual |
| 9 | L | -55.9 | -47.7 | -9.3 | 1 | 11 | FrontoParietal |
| 10 | L | -32 | -29.3 | 15.6 | 3 | 3 | Auditory |
| 11 | L | -29.3 | 5.3 | -27.4 | 4 | 1 | None |
| 12 | L | -6.1 | -26 | 28.5 | 4 | 1 | CinguloParietal |
| 13 | L | -14.4 | -57.8 | 18.4 | 1 | 4 | RetrosplenialTemporal |
| 14 | L | -8.8 | -49.8 | 4.2 | 1 | 4 | RetrosplenialTemporal |
| 15 | L | -11.3 | -83.2 | 3.9 | 5 | 8 | Visual |
| 16 | L | -22 | -58.1 | 1.5 | 5 | 8 | Visual |
| 17 | L | -9.6 | -58 | 3 | 5 | 8 | Visual |
| 18 | L | -29 | -35.9 | -8.3 | 4 | 1 | None |
| 19 | L | -18.5 | -39.2 | -1.1 | 4 | 1 | None |
| 20 | L | -16.7 | -46 | -3.7 | 5 | 8 | Visual |
| 21 | L | -16.6 | -36.1 | 42.7 | 2 | 2 | CinguloOperc |
| 22 | L | -9.4 | -0.1 | 42.9 | 2 | 2 | CinguloOperc |
| 23 | L | -3.8 | 12.1 | 64.6 | 1 | 9 | VentralAttn |
| 24 | L | -5.5 | 29.3 | 44 | 1 | 9 | FrontoParietal |
| 25 | L | -5.6 | 42.2 | 35.1 | 1 | 9 | Default |
| 26 | L | -1.7 | -17.7 | 39.1 | 1 | 4 | Default |
| 27 | L | -8.4 | 14.6 | 33.8 | 2 | 2 | CinguloOperc |
| 28 | L | -9 | 25.3 | 27.7 | 2 | 2 | CinguloOperc |
| 29 | L | -10 | 33.9 | 21.5 | 2 | 2 | Salience |
| 30 | L | -10.7 | -47.5 | 60.3 | 3 | 12 | SMhand |
| 31 | L | -15.6 | -33.1 | 66.1 | 3 | 12 | SMhand |
| 32 | L | -10.9 | -29.3 | 69.5 | 3 | 12 | SMhand |
| 33 | L | -6.6 | -20.4 | 74.2 | 3 | 12 | SMhand |
| 34 | L | -8 | -8.7 | 62.9 | 3 | 7 | CinguloOperc |
| 35 | L | -10.8 | -41.1 | 64.9 | 3 | 12 | SMhand |
| 36 | L | -5 | -28.2 | 60.4 | 3 | 12 | SMhand |
| 37 | L | -5.4 | -15.9 | 48.8 | 3 | 12 | SMhand |
| 38 | L | -35.8 | -29.7 | 54.5 | 3 | 7 | SMhand |
| 39 | L | -41.5 | -12.5 | 50.4 | 3 | 3 | SMmouth |
| 40 | L | -42.1 | -4.5 | 47.3 | 2 | 14 | CinguloOperc |
| 41 | L | -27.3 | -6.8 | 46.3 | 2 | 14 | DorsalAttn |
| 42 | L | -27.3 | 1.9 | 52.9 | 2 | 14 | DorsalAttn |
| 43 | L | -19.8 | 6.4 | 55.7 | 2 | 14 | DorsalAttn |
| 44 | L | -19.5 | 30.1 | 45.5 | 1 | 4 | Default |
| 45 | L | -36.8 | -22.8 | 61.9 | 3 | 7 | SMhand |
| 46 | L | -20.5 | -24.9 | 64.5 | 3 | 12 | SMhand |
| 47 | L | -23.4 | -13.8 | 64.2 | 3 | 7 | SMhand |
| 48 | L | -17.2 | -8.6 | 67.9 | 2 | 14 | SMhand |
| 49 | L | -21.3 | -0.2 | 62.7 | 2 | 14 | DorsalAttn |
| 50 | L | -28.6 | -44.7 | 61.7 | 3 | 7 | SMhand |
| 51 | L | -31.1 | -48.9 | 47.1 | 2 | 5 | DorsalAttn |
| 52 | L | -42.9 | -45 | 43 | 2 | 5 | DorsalAttn |
| 53 | L | -51.5 | -11.9 | 29.7 | 3 | 3 | SMmouth |
| 54 | L | -54.1 | -21.3 | 40.8 | 3 | 7 | SMhand |
| 55 | L | -51.7 | -30.9 | 39.9 | 2 | 5 | DorsalAttn |
| 56 | L | -35.2 | -35.3 | 42 | 3 | 7 | SMhand |
| 57 | L | -27.5 | -37.2 | 61.4 | 3 | 7 | SMhand |
| 58 | L | -47.2 | -31.4 | 54.8 | 3 | 7 | SMhand |
| 59 | L | -46.1 | -17.8 | 52.7 | 3 | 7 | SMmouth |
| 60 | L | -44.8 | -54 | 14.6 | 4 | 1 | VentralAttn |
| 61 | L | -51.6 | -55.9 | 11.4 | 1 | 13 | VentralAttn |
| 62 | L | -48.1 | -40 | 2.4 | 1 | 13 | VentralAttn |
| 63 | L | -57.7 | -40.6 | 35.8 | 2 | 5 | CinguloOperc |
| 64 | L | -46.3 | -41.4 | 25.9 | 2 | 2 | Auditory |
| 65 | L | -35.8 | -33.5 | 19.9 | 3 | 3 | Auditory |
| 66 | L | -52.7 | -20.6 | 5.4 | 3 | 3 | Auditory |
| 67 | L | -59.6 | -38.5 | 16.5 | 3 | 3 | Auditory |
| 68 | L | -58.7 | -29.9 | 11.1 | 3 | 3 | Auditory |
| 69 | L | -40.6 | -38.3 | 14.5 | 3 | 3 | Auditory |
| 70 | L | -33.7 | -21.8 | 9.9 | 3 | 3 | Auditory |
| 71 | L | -38.7 | -16 | -5.3 | 2 | 2 | CinguloOperc |
| 72 | L | -39.1 | -1.6 | -12.2 | 2 | 2 | CinguloOperc |
| 73 | L | -33.6 | 17.2 | -31.5 | 1 | 13 | None |
| 74 | L | -43.6 | 36.3 | 8.5 | 2 | 5 | DorsalAttn |
| 75 | L | -50 | 20.8 | 10.6 | 1 | 9 | VentralAttn |
| 76 | L | -37.7 | 2.9 | 11.7 | 2 | 2 | CinguloOperc |
| 77 | L | -37.2 | -14 | 19.4 | 3 | 3 | Auditory |
| 78 | L | -40.3 | 50.4 | -4.8 | 1 | 9 | FrontoParietal |
| 79 | L | -47.2 | 39 | -9.1 | 1 | 9 | VentralAttn |
| 80 | L | -29.1 | 20.5 | -14 | 1 | 9 | VentralAttn |
| 81 | L | -36.6 | 1.4 | 6.4 | 2 | 2 | CinguloOperc |
| 82 | L | -37.3 | 8.9 | -0.9 | 2 | 2 | CinguloOperc |
| 83 | L | -32.5 | 17.2 | -7.8 | 4 | 1 | Salience |
| 84 | L | -28.8 | 23.7 | 8.4 | 2 | 2 | CinguloOperc |
| 85 | L | -44.3 | 33.2 | -7.2 | 1 | 9 | VentralAttn |
| 86 | L | -45.4 | 28.8 | 0.8 | 1 | 9 | VentralAttn |
| 87 | L | -20.4 | -64.6 | 51.4 | 5 | 6 | DorsalAttn |
| 88 | L | -25.8 | -65 | 32.2 | 5 | 6 | DorsalAttn |
| 89 | L | -12.7 | -64.9 | 31.8 | 4 | 1 | CinguloParietal |
| 90 | L | -13.7 | -77.4 | 26.6 | 5 | 8 | Visual |
| 91 | L | -9.9 | -56.9 | 59.8 | 2 | 14 | DorsalAttn |
| 92 | L | -7.1 | -63.7 | 54.9 | 2 | 14 | DorsalAttn |
| 93 | L | -10.9 | -73.4 | 42.9 | 4 | 1 | CinguloParietal |
| 94 | L | -39.3 | -73.9 | 38.3 | 1 | 4 | Default |
| 95 | L | -30 | -74.1 | 36.1 | 1 | 11 | DorsalAttn |
| 96 | L | -34.1 | -61 | 42.4 | 1 | 11 | FrontoParietal |
| 97 | L | -31.3 | -84.2 | 9 | 5 | 6 | Visual |
| 98 | L | -34.2 | -86.6 | -0.5 | 5 | 6 | Visual |
| 99 | L | -43.4 | -67.6 | 9.7 | 5 | 6 | Visual |
| 100 | L | -46.2 | -57.7 | -7.9 | 5 | 6 | DorsalAttn |
| 101 | L | -59.8 | -4.1 | 8.8 | 3 | 3 | CinguloOperc |
| 102 | L | -52.2 | -14.1 | 15.2 | 3 | 3 | Auditory |
| 103 | L | -55.1 | -32.3 | 23 | 2 | 2 | CinguloOperc |
| 104 | L | -50.6 | -22.4 | 19.2 | 3 | 3 | Auditory |
| 105 | L | -58.8 | -23.9 | 31 | 2 | 5 | CinguloOperc |
| 106 | L | -45.2 | 2.7 | 32.4 | 2 | 5 | DorsalAttn |
| 107 | L | -34.7 | 5.6 | 34 | 2 | 5 | DorsalAttn |
| 108 | L | -43 | 19.4 | 33.5 | 1 | 9 | FrontoParietal |
| 109 | L | -40.2 | 23.6 | 23.3 | 1 | 9 | FrontoParietal |
| 110 | L | -37.6 | 38.4 | 17.2 | 2 | 5 | DorsalAttn |
| 111 | L | -51.8 | -0.6 | 5 | 2 | 2 | CinguloOperc |
| 112 | L | -48.6 | 7.5 | 11.1 | 2 | 5 | CinguloOperc |
| 113 | L | -41.6 | 8.7 | 22.2 | 2 | 5 | DorsalAttn |
| 114 | L | -27.5 | 53.6 | 0 | 4 | 1 | Default |
| 115 | L | -23.4 | 61 | -6.8 | 4 | 1 | None |
| 116 | L | -5.9 | 54.8 | -11.3 | 1 | 4 | Default |
| 117 | L | -6.8 | 38.2 | -9.4 | 1 | 4 | Default |
| 118 | L | -31.8 | 2.6 | -16.8 | 4 | 1 | None |
| 119 | L | -34.7 | 35.6 | -9.6 | 4 | 1 | None |
| 120 | L | -22.5 | 32.1 | -13.6 | 4 | 1 | None |
| 121 | L | -23.8 | 52.2 | -12.8 | 4 | 1 | None |
| 122 | L | -17.3 | 46.6 | -17.9 | 4 | 1 | None |
| 123 | L | -13.3 | 24 | -16.4 | 4 | 1 | None |
| 124 | L | -8.9 | 45.5 | -20.8 | 4 | 1 | None |
| 125 | L | -2.5 | 33.8 | -26.2 | 4 | 1 | None |
| 126 | L | -63.2 | -28.7 | -7.2 | 1 | 9 | Default |
| 127 | L | -53.1 | -11.4 | -16 | 1 | 13 | Default |
| 128 | L | -53.2 | -13 | -29.2 | 1 | 9 | None |
| 129 | L | -44.6 | 9 | -37 | 1 | 13 | None |
| 130 | L | -33.8 | -33.2 | -15.4 | 1 | 4 | RetrosplenialTemporal |
| 131 | L | -28.8 | -58.8 | -9.1 | 5 | 8 | Visual |
| 132 | L | -34.4 | -63.9 | -15.7 | 5 | 6 | Visual |
| 133 | L | -55.1 | -39.6 | -16.2 | 1 | 11 | None |
| 134 | L | -32 | -3.9 | -45.2 | 4 | 1 | None |
| 135 | L | -38.6 | -13 | -26.9 | 4 | 1 | None |
| 136 | L | -34.3 | -43.8 | -21.6 | 5 | 6 | Visual |
| 137 | L | -5.4 | -88 | 18.6 | 5 | 8 | Visual |
| 138 | L | -8.6 | -77.5 | -3.5 | 5 | 8 | Visual |
| 139 | L | -41.2 | -72.1 | -5.9 | 5 | 6 | Visual |
| 140 | L | -25.2 | -97.2 | -7.9 | 5 | 6 | Visual |
| 141 | L | -22.6 | -81.7 | -11.7 | 5 | 6 | Visual |
| 142 | L | -20.5 | -12.6 | -23.7 | 1 | 4 | None |
| 143 | L | -22.5 | -37.1 | -15 | 1 | 4 | RetrosplenialTemporal |
| 144 | L | -22 | -21.9 | -17.4 | 1 | 4 | None |
| 145 | L | -15.9 | 48.6 | 37.2 | 1 | 9 | Default |
| 146 | L | -19.5 | 56.3 | 27.5 | 1 | 9 | Default |
| 147 | L | -26.6 | 46.8 | 20.9 | 2 | 5 | CinguloOperc |
| 148 | L | -21.3 | 63.1 | 1.9 | 1 | 4 | FrontoParietal |
| 149 | L | -28.6 | 50.9 | 10.1 | 4 | 1 | FrontoParietal |
| 150 | L | -6.5 | 54.7 | 18.1 | 1 | 4 | Default |
| 151 | L | -15.7 | 64.7 | 13.7 | 1 | 4 | Default |
| 152 | L | -6 | 44.9 | 6.3 | 1 | 10 | Default |
| 153 | L | -28.8 | 38.3 | 28.2 | 2 | 5 | CinguloOperc |
| 154 | L | -26.2 | 26.6 | 38.8 | 1 | 4 | Default |
| 155 | L | -35.7 | 33.1 | 32 | 2 | 5 | DorsalAttn |
| 156 | L | -29.3 | 16.8 | 50.7 | 1 | 9 | Default |
| 157 | L | -41.7 | 16.1 | 47.5 | 1 | 9 | Default |
| 158 | L | -38.7 | 4.8 | 48.4 | 1 | 9 | VentralAttn |
| 159 | L | -50.8 | 6.9 | -20.1 | 1 | 13 | None |
| 160 | L | -54.4 | -1.4 | -0.7 | 3 | 3 | Auditory |
| 161 | L | -59 | -18 | -3 | 1 | 13 | VentralAttn |
| 162 | R | 12.3 | -51.6 | 34.5 | 1 | 4 | Default |
| 163 | R | 20.8 | -48.2 | 66.1 | 3 | 7 | SMhand |
| 164 | R | 49.6 | -7.4 | 36.1 | 3 | 3 | SMmouth |
| 165 | R | 11.9 | 21.9 | 59.9 | 1 | 10 | Default |
| 166 | R | 22 | -84.6 | 23.7 | 5 | 8 | Visual |
| 167 | R | 47.9 | -42.5 | 41.5 | 1 | 11 | FrontoParietal |
| 168 | R | 38.1 | 45.9 | 7.7 | 2 | 5 | FrontoParietal |
| 169 | R | 22.3 | -46.5 | -9.9 | 5 | 8 | Visual |
| 170 | R | 59.7 | -41 | -10.9 | 1 | 11 | FrontoParietal |
| 171 | R | 33.6 | -22.3 | 13 | 3 | 3 | Auditory |
| 172 | R | 32.5 | 13.6 | -30.5 | 4 | 1 | None |
| 173 | R | 7.6 | -27 | 28.4 | 4 | 1 | CinguloParietal |
| 174 | R | 13.8 | -54.1 | 10.9 | 1 | 4 | RetrosplenialTemporal |
| 175 | R | 15.5 | -74.1 | 9.4 | 5 | 8 | Visual |
| 176 | R | 19.6 | -45.3 | -4.4 | 5 | 8 | Visual |
| 177 | R | 15.6 | -59.6 | -5 | 5 | 8 | Visual |
| 178 | R | 24.9 | -35.9 | -4.8 | 4 | 1 | None |
| 179 | R | 19.4 | -29.9 | -9.7 | 4 | 1 | None |
| 180 | R | 16.2 | -33.1 | 43.2 | 2 | 2 | CinguloOperc |
| 181 | R | 6.7 | 5 | 55.9 | 2 | 2 | CinguloOperc |
| 182 | R | 7 | 25.7 | 47.3 | 1 | 11 | FrontoParietal |
| 183 | R | 8.4 | 34.7 | 22.6 | 4 | 1 | Salience |
| 184 | R | 7.7 | 44.1 | 5.5 | 1 | 10 | Default |
| 185 | R | 8.6 | 4.2 | 40.1 | 2 | 2 | CinguloOperc |
| 186 | R | 3 | -19.6 | 37.9 | 1 | 4 | Default |
| 187 | R | 8.8 | 10.8 | 45.9 | 2 | 2 | CinguloOperc |
| 188 | R | 6 | 21.8 | 32.4 | 2 | 2 | CinguloOperc |
| 189 | R | 10.3 | -57.3 | 58.3 | 2 | 14 | DorsalAttn |
| 190 | R | 16.5 | -32.8 | 67.7 | 3 | 12 | SMhand |
| 191 | R | 4.8 | -27.1 | 64.8 | 3 | 12 | SMhand |
| 192 | R | 16.2 | 0.8 | 67.5 | 2 | 2 | CinguloOperc |
| 193 | R | 11.9 | -40.7 | 67 | 3 | 12 | SMhand |
| 194 | R | 5.1 | -17.1 | 51.6 | 3 | 12 | SMhand |
| 195 | R | 6.8 | -8.1 | 50.9 | 3 | 12 | SMhand |
| 196 | R | 8 | -6.2 | 63.7 | 2 | 2 | CinguloOperc |
| 197 | R | 42.3 | -11 | 47.3 | 3 | 3 | SMmouth |
| 198 | R | 42.5 | -2.3 | 47.2 | 2 | 14 | CinguloOperc |
| 199 | R | 29.2 | 1.9 | 52.4 | 2 | 14 | DorsalAttn |
| 200 | R | 21.9 | 21 | 46.2 | 1 | 4 | Default |
| 201 | R | 38.1 | -22.4 | 60.3 | 3 | 7 | SMhand |
| 202 | R | 19.7 | -25 | 65.2 | 3 | 12 | SMhand |
| 203 | R | 29.9 | -7.8 | 47.4 | 2 | 14 | DorsalAttn |
| 204 | R | 12.4 | -28.3 | 69.6 | 3 | 12 | SMhand |
| 205 | R | 29.2 | -13.5 | 64.2 | 3 | 7 | SMhand |
| 206 | R | 17 | -16.9 | 70.9 | 3 | 12 | SMhand |
| 207 | R | 20.9 | -6.4 | 65 | 2 | 14 | SMhand |
| 208 | R | 22.6 | 5.6 | 57.6 | 2 | 14 | DorsalAttn |
| 209 | R | 29.5 | -42.5 | 60.4 | 3 | 7 | SMhand |
| 210 | R | 34.2 | -40.6 | 51.6 | 4 | 1 | SMhand |
| 211 | R | 38.8 | -42.6 | 40.4 | 2 | 5 | DorsalAttn |
| 212 | R | 53.9 | -8.3 | 26.1 | 3 | 3 | SMmouth |
| 213 | R | 39.6 | -31.5 | 39.7 | 3 | 7 | SMhand |
| 214 | R | 28 | -34.8 | 63.1 | 3 | 12 | SMhand |
| 215 | R | 39.2 | -34.6 | 57.5 | 3 | 7 | SMhand |
| 216 | R | 37.3 | -25.9 | 50.9 | 3 | 7 | SMhand |
| 217 | R | 48.7 | -26.1 | 52.2 | 3 | 7 | SMhand |
| 218 | R | 47.8 | -15.1 | 49.3 | 3 | 7 | SMmouth |
| 219 | R | 57.5 | -40.3 | 34.7 | 2 | 5 | CinguloOperc |
| 220 | R | 48.9 | -53 | 28.6 | 1 | 10 | Default |
| 221 | R | 57.5 | -45.3 | 9 | 1 | 13 | VentralAttn |
| 222 | R | 60.9 | -38.7 | 1.7 | 1 | 10 | VentralAttn |
| 223 | R | 54.9 | -27 | 29.6 | 2 | 14 | CinguloOperc |
| 224 | R | 36.4 | -30.7 | 19.4 | 3 | 3 | Auditory |
| 225 | R | 62.5 | -25.6 | -5.5 | 1 | 10 | Default |
| 226 | R | 57.1 | -17 | -2.6 | 1 | 13 | VentralAttn |
| 227 | R | 53.8 | -15.8 | 5.2 | 3 | 3 | Auditory |
| 228 | R | 47.4 | -39.6 | 13.2 | 1 | 13 | VentralAttn |
| 229 | R | 45.5 | -37.3 | 3.4 | 1 | 13 | VentralAttn |
| 230 | R | 59.2 | -38.6 | 14.6 | 3 | 3 | Auditory |
| 231 | R | 48.5 | -26.5 | -0.1 | 1 | 13 | VentralAttn |
| 232 | R | 61.7 | -24 | 1.3 | 3 | 3 | Auditory |
| 233 | R | 60 | -25.2 | 10.2 | 3 | 3 | Auditory |
| 234 | R | 38.8 | -14.4 | -5 | 2 | 2 | CinguloOperc |
| 235 | R | 39.7 | 1.2 | -13.1 | 2 | 2 | CinguloOperc |
| 236 | R | 36.8 | 37.8 | 13.1 | 2 | 5 | DorsalAttn |
| 237 | R | 52.5 | 23.7 | 10.3 | 1 | 10 | VentralAttn |
| 238 | R | 36.7 | 5.2 | 12.7 | 2 | 2 | CinguloOperc |
| 239 | R | 38.4 | -12.2 | 20 | 3 | 3 | Auditory |
| 240 | R | 42.8 | 48.3 | -5.1 | 1 | 11 | FrontoParietal |
| 241 | R | 48.1 | 38.3 | -9.2 | 1 | 10 | VentralAttn |
| 242 | R | 45.2 | 30.7 | -5.6 | 1 | 10 | VentralAttn |
| 243 | R | 27.4 | 19.7 | -14.9 | 1 | 10 | VentralAttn |
| 244 | R | 36.6 | -10 | 12.4 | 3 | 3 | Auditory |
| 245 | R | 39.6 | 10.4 | -1.6 | 2 | 2 | CinguloOperc |
| 246 | R | 36.5 | 5.7 | 6 | 2 | 2 | CinguloOperc |
| 247 | R | 30.6 | 22.8 | -4.7 | 2 | 2 | Salience |
| 248 | R | 33.7 | 22.6 | 3.7 | 2 | 2 | CinguloOperc |
| 249 | R | 34 | 24.4 | 10 | 2 | 2 | CinguloOperc |
| 250 | R | 48.1 | 38.4 | 2.4 | 2 | 5 | DorsalAttn |
| 251 | R | 26.8 | -55 | 54.2 | 5 | 6 | Visual |
| 252 | R | 23 | -66.4 | 51.8 | 5 | 6 | DorsalAttn |
| 253 | R | 32.3 | -63.6 | 33.8 | 5 | 6 | DorsalAttn |
| 254 | R | 15.6 | -69.5 | 39.6 | 4 | 1 | CinguloParietal |
| 255 | R | 17.6 | -78.3 | 34 | 5 | 8 | Visual |
| 256 | R | 7.7 | -85.6 | 31.6 | 5 | 8 | Visual |
| 257 | R | 7.4 | -69.3 | 49.9 | 2 | 14 | Default |
| 258 | R | 35.4 | -77.1 | 21.1 | 5 | 6 | Visual |
| 259 | R | 46.5 | -67.3 | 36.2 | 1 | 11 | Default |
| 260 | R | 41.5 | -53.5 | 44 | 1 | 11 | FrontoParietal |
| 261 | R | 35.7 | -56.7 | 45.2 | 1 | 11 | FrontoParietal |
| 262 | R | 33.5 | -48.2 | 49.4 | 2 | 5 | DorsalAttn |
| 263 | R | 31.7 | -85.7 | 2.4 | 5 | 6 | Visual |
| 264 | R | 43.8 | -67.2 | 2 | 5 | 6 | Visual |
| 265 | R | 47.3 | -52.4 | -11.7 | 5 | 6 | Visual |
| 266 | R | 57 | -53.8 | -1.1 | 2 | 5 | DorsalAttn |
| 267 | R | 49 | -54.5 | 8.8 | 5 | 6 | Visual |
| 268 | R | 60.9 | -2.2 | 10.7 | 3 | 3 | Auditory |
| 269 | R | 54.2 | -13.6 | 16.9 | 3 | 3 | Auditory |
| 270 | R | 53 | -22.7 | 39.1 | 3 | 7 | SMhand |
| 271 | R | 47.3 | 2 | 37.6 | 2 | 5 | DorsalAttn |
| 272 | R | 37.8 | 28.7 | 35.6 | 1 | 11 | FrontoParietal |
| 273 | R | 41.8 | 29.1 | 21.6 | 1 | 11 | FrontoParietal |
| 274 | R | 50.1 | 3 | 3.9 | 2 | 2 | CinguloOperc |
| 275 | R | 46.6 | 7.8 | 19.3 | 2 | 5 | DorsalAttn |
| 276 | R | 38.6 | 18.8 | 25.5 | 4 | 1 | FrontoParietal |
| 277 | R | 28.4 | 57 | -5.1 | 1 | 11 | FrontoParietal |
| 278 | R | 4.8 | 65.1 | -7.1 | 1 | 4 | Default |
| 279 | R | 7.2 | 48.4 | -10.1 | 1 | 4 | Default |
| 280 | R | 2.9 | 18.7 | -23.2 | 4 | 1 | None |
| 281 | R | 35.1 | 37.3 | -8.4 | 4 | 1 | None |
| 282 | R | 25.4 | 8.9 | -15.7 | 4 | 1 | None |
| 283 | R | 21.2 | 30.3 | -15.2 | 4 | 1 | None |
| 284 | R | 21.6 | 51.1 | -14.1 | 4 | 1 | None |
| 285 | R | 11.9 | 25.7 | -24.8 | 4 | 1 | None |
| 286 | R | 13.5 | 20.3 | -15.2 | 4 | 1 | None |
| 287 | R | 10.9 | 39.1 | -19.7 | 4 | 1 | None |
| 288 | R | 2.2 | 39 | -25.6 | 4 | 1 | None |
| 289 | R | 62.3 | -26.4 | -16 | 4 | 1 | None |
| 290 | R | 57.5 | -7.4 | -16.4 | 1 | 4 | Default |
| 291 | R | 54.7 | -7.8 | -26.9 | 1 | 10 | None |
| 292 | R | 45.2 | 13.6 | -30.1 | 1 | 13 | None |
| 293 | R | 31.2 | -45.6 | -5.8 | 5 | 8 | Visual |
| 294 | R | 34.6 | -35.6 | -12.3 | 4 | 1 | RetrosplenialTemporal |
| 295 | R | 34.6 | -23.9 | -20.4 | 4 | 1 | RetrosplenialTemporal |
| 296 | R | 20.1 | -21.4 | -21.5 | 4 | 1 | None |
| 297 | R | 28 | -0.4 | -37.3 | 4 | 1 | None |
| 298 | R | 26.9 | -69.1 | -6.6 | 5 | 6 | Visual |
| 299 | R | 34.9 | -44 | -20 | 5 | 6 | Visual |
| 300 | R | 36.8 | 7.7 | -37.9 | 4 | 1 | None |
| 301 | R | 54.5 | -9.6 | -37 | 4 | 1 | None |
| 302 | R | 56.4 | -27 | -19.4 | 4 | 1 | None |
| 303 | R | 31.1 | 2.2 | -46.1 | 4 | 1 | None |
| 304 | R | 39.5 | -11.9 | -29.7 | 4 | 1 | None |
| 305 | R | 31.6 | -9.4 | -35.5 | 4 | 1 | None |
| 306 | R | 43.4 | -24.1 | -20.8 | 4 | 1 | None |
| 307 | R | 13.8 | -92.3 | 14.7 | 5 | 8 | Visual |
| 308 | R | 10.5 | -73.8 | -1.5 | 5 | 8 | Visual |
| 309 | R | 20.4 | -87.3 | -6.6 | 5 | 6 | Visual |
| 310 | R | 5.1 | -80.2 | 23.1 | 5 | 8 | Visual |
| 311 | R | 14.6 | -70.3 | 23.3 | 5 | 8 | Visual |
| 312 | R | 19.5 | -10.8 | -24.9 | 4 | 1 | None |
| 313 | R | 24.5 | -36.2 | -13.2 | 1 | 4 | RetrosplenialTemporal |
| 314 | R | 30.4 | -18.8 | -19.4 | 4 | 1 | None |
| 315 | R | 21 | 32.8 | 42.1 | 1 | 4 | Default |
| 316 | R | 21.4 | 42.8 | 35.1 | 1 | 10 | Default |
| 317 | R | 24.4 | 50.8 | 24.3 | 2 | 5 | CinguloOperc |
| 318 | R | 31.3 | 39.7 | 25.6 | 2 | 5 | CinguloOperc |
| 319 | R | 23.5 | 59.1 | 4.9 | 1 | 11 | FrontoParietal |
| 320 | R | 30.9 | 52.2 | 9.9 | 1 | 11 | FrontoParietal |
| 321 | R | 16 | 61 | 19.8 | 1 | 10 | Default |
| 322 | R | 8.2 | 53.8 | 14 | 1 | 10 | Default |
| 323 | R | 5.9 | 54.9 | 29.4 | 1 | 10 | Default |
| 324 | R | 13.8 | 46.7 | 42.1 | 1 | 10 | Default |
| 325 | R | 6.8 | 44.5 | 34.8 | 1 | 10 | Default |
| 326 | R | 30.6 | 18.9 | 48.7 | 1 | 4 | Default |
| 327 | R | 42.4 | 19.5 | 48.2 | 1 | 11 | FrontoParietal |
| 328 | R | 38.9 | 9.6 | 42.7 | 1 | 10 | FrontoParietal |
| 329 | R | 39.7 | -22.5 | 2.6 | 4 | 1 | Auditory |
| 330 | R | 55.8 | 2 | -2 | 3 | 3 | Auditory |
| 331 | R | 54.4 | 1.1 | -12.9 | 1 | 13 | Default |
| 332 | R | 57.1 | -6.3 | -7.7 | 1 | 13 | VentralAttn |
| 333 | R | 46.6 | -21.5 | -8.5 | 1 | 13 | VentralAttn |
